# Liposomal ellagic acid enhances the regenerative potential of ADMSC-laden nanofibrous PCL scaffolds in a rat model of spinal cord injury

**DOI:** 10.1038/s41598-025-15789-w

**Published:** 2025-08-18

**Authors:** Arman Abroumand Gholami, Shokoufeh Rahmani, Payam Moharreri, Esmael Amirazodi, Amir Mahdi Molavi, Tahere Mokhtari, Fatemeh Tahmasebi, Amirhossein Rabiei Rad, Hamideh Babaloo

**Affiliations:** 1 Nervous System Stem Cell Research Center, Semnan University of Medical Sciences, Semnan, Iran; 2 Tissue Engineering Research Group (TERG), Department of Anatomy and Cell Biology, School of Medicine, Mashhad University of Medical Sciences, Mashhad, Iran; 3 Neuroscience Research Center, Mashhad University of Medical Sciences, Mashhad, Iran; 4 School of Pharmacy, Shiraz University of Medical Sciences, Shiraz, Iran; 5 Department of Neurology, Ahwaz Jundishapur University of Medical Sciences, Ahvaz, Iran; 6 Department of Materials Research, Iranian Academic Center for Education, Culture and Research, Khorasan Razavi Branch, Mashhad, Iran; 7 Department of Pathology, Division of Experimental Pathology, School of Medicine, University of Pittsburgh, Pittsburgh, PA USA; 8 Faculty of Health Sciences, Department of Physiotherapy and Rehabilitation, Tokat Gaziosmanpaşa University, Tokat, Turkey; 9 Biotechnology Research Center, International Campus, Yazd Reproductive Sciences Institute, Shahid Sadoughi University of Medical Sciences, Yazd, Iran; 10 School of Advanced Medical Technologies, Shahid Sadoughi University of Medical Sciences, Yazd, Iran; 11 Present Address: Department of Anatomy, Saveh University of Medical Sciences, Saveh, Iran

**Keywords:** Antioxidant, Mesenchymal stem cell, Multiwall carbon nanotube, Nanoliposome, Polycaprolactone, Neurological disorders, Trauma, Biochemistry, Biological techniques, Biotechnology, Cell biology, Chemical biology, Molecular biology, Neuroscience, Stem cells, Medical research, Molecular medicine, Materials science, Nanoscience and technology

## Abstract

Spinal cord injury (SCI) leads to myelin breakdown and extensive neuronal loss around the injury site due to increased oxidative stress. This study aims to develop a comprehensive platform incorporating scaffolds, therapeutic agents, and stem cells to restore structures and pathways in SCI. Scaffolds were created through the electrospinning of a PCL/functionalized multi-walled carbon nanotube (f-MWCNTs) composite, which was then coated with liposomal ellagic acid (EA@lip) and seeded with adipose-derived mesenchymal stem cells (ADMSCs). The optimal drug concentration was determined by conducting MTT and DPPH assays through three different time points. After assessing the biocompatibility and anti-inflammatory properties of the scaffolds for ADMSCs, the implant was tested in a rat model of dorsal hemisection. The female Wistar rats were divided into six groups (*n* = 10): Sham, SCI, SCI + PCL/f-MWCNTs (PCs), SCI + scaffolds + EA@lip (PC/N), SCI + scaffolds + ADMSCs (PC/C), and SCI + scaffolds + EA@lip + ADMSCs (PC/N/C). In the second week, biochemical analyses were conducted to evaluate oxidative stress in the animals’ blood. Throughout the study, the motor function of the animals was monitored. After six weeks, the rats were subjected to real-time PCR and histological analysis, utilizing Cresyl Violet/Luxol Fast Blue staining and evaluating the expression of the genes COX2, GPX1, MBP, and Slc17a6/7. Liposomal encapsulation efficiency was measured to be 33%. The results revealed that EA@lip had the desired size, zeta potential, and lipid concentration. Transmission electron microscopy revealed that f-MWCNTs were well-aligned along nanofibers. EA@lip dramatically enhanced the hydrophilicity of the scaffolds. The MTT assay, DAPI staining, and FE-SEM images confirmed the successful implantation, proliferation, adhesion, and survival of ADMSCs on the liposome-coated scaffold. Additionally, in vitro oxidative stress tests indicated that this platform exhibited superior antioxidant and anti-inflammatory effects for ADMSCs. Histological assessments revealed that the hybrid platform facilitated the regeneration of myelin and neurons, correlating with improved blood levels of oxidative markers. Furthermore, real-time PCR results demonstrated a decrease in COX2 expression and an increase in GPX1, MBP, and Slc17a6/7 expression due to the platform. The findings suggest that the combination of ADMSCs with EA@lip-coated PCL/f-MWCNT scaffolds hold significant promise for applications in spinal cord regeneration.

## Introduction

Spinal cord injury (SCI) is a severe condition that leads to long-term or permanent loss of sensory and motor function below the injury site^1,2^. This leads to neuronal death and myelin degeneration, which are further exacerbated by secondary injury mechanisms that elevate oxidative stress (OS), promote inflammation, induce neuronal and oligodendrocyte apoptosis, and contribute to cystic cavity formation^3–8^. These pathological events progressively alter neuronal activity and disrupt the structural integrity of the damaged tissue by enhancing lipid peroxidation (LPO) and generating reactive aldehydes^9–12^. Notably, experimental studies have demonstrated that therapeutic approaches targeting oxidative stress, by reducing reactive oxygen species (ROS) and LPO, can significantly improve histological and functional outcomes following SCI^3,13^. This therapeutic potential is attributed to the high content of unsaturated fatty acids in spinal cord neurons, which renders them particularly vulnerable to LPO-induced damage^14,15^. Consequently, considerable attention has been directed toward the development of novel treatment strategies for SCI, with particular focus on bioactive compounds and cell-based therapies as promising antioxidant interventions^4,7,16–19^.

There has been a recent focus on electrospun fiber scaffolds as a potential option for treating SCIs. These nanofibers have advantages over traditional biological materials in terms of their structure and porosity. They resemble the natural extracellular matrix, which can help with cell attachment and development^20–22^. To facilitate spinal cord regeneration, various types of polymers, particularly polyesters, have been developed for scaffolds. Polyesters such as polycaprolactone (PCL) possess desirable qualities, such as strength, biocompatibility, and biodegradability^23^. However, the low hydrophilicity of PCL limits its effectiveness in terms of cell adhesion, proliferation, migration, and differentiation^20,24^. One solution involves increasing the wettability of PCL fibers by incorporating hydrophilic materials. Multiwalled carbon nanotubes (MWCNTs) have been widely used in regenerating different tissues. Their needle-like structure enhances scaffold strength, and their inner hollow space and high surface area allow for binding with bioactive molecules^25^. Owing to their conductivity, CNTs also have good compatibility with interneuronal cellular communication^26^. However, their hydrophobic nature leads to severe aggregation in aqueous solutions, limiting their biological applications. To overcome this, functionalizing of MWCNTs (f-MWCNTs) is necessary^27^.

In addition to providing a suitable substrate for axon growth and neuronal recovery, biologically active substances play a crucial role for controlling the oxidative environment of SCI^3,6^. By incorporating an antioxidant drug into a polymer system near the target tissue, it is possible to increase the local drug concentration and reduce tissue exposure to stress^20^. Ellagic acid (EA), a polyphenol, has gained significant attention due to its various physiological and pharmacological effects^28–30^. EA has been found to regulate cyclooxygenase (COX) enzyme activity, exhibiting strong anti-inflammatory properties in addition to modulating oxidative status and LPO^31–34^. However, EA suffers from poor water solubility and limited therapeutic efficacy and clinical applicability because of its high log P^35^. This issue can be resolved by encapsulating the drug into liposomes. They protect bioactive compounds from various environmental factors, such as enzymatic degradation, oxidation, molecular interactions, and pH changes^36^. Liposomes are not cytotoxic and do not stimulate the immune system^37^. Moreover, liposomes can carry both hydrophilic and hydrophobic drugs and enhance surface hydrophilicity to support cell attachment^36^. Microencapsulation of EA within nanoliposomes provides better absorption for tissue regeneration, longer release, and increased drug half-life with regulated distribution^35^.

The need for cell therapy is intensified by the inability of the spinal cord to heal itself and replace lost native cells^18^. Recent data have shown that mesenchymal stem cell (MSC) therapy is effective and safe for improving spinal cord dysfunction^4^. However, there are ethical concerns and safety issues associated with the various cell sources used in experimental research, such as bone marrow-derived MSCs, hematopoietic stem/progenitor cells, and cells of embryonic origin^38^. The use of adipose-derived MSCs (ADMSCs) has distinct advantages, including minimally invasive harvesting and an unlimited supply from in vitro culture. Compared with MSCs derived from bone marrow, ADMSCs exhibit stronger antioxidant, anti-inflammatory, and immunomodulatory functions^7,16,39^. These properties strengthen the antioxidant system, leading to the removal of free radicals and the self-production of reactive aldehydes, which can increase neuron activity after injury^4^.

Thus, our hypothesis is that by implanting ADMSCs on a modified scaffold that targets the suppressed oxidative state through enhancement of the antioxidant system by reducing reactive oxygen species (ROS) and LPO, we can preserve the integrity of microanatomical structures and facilitate the recovery of motor function following SCI.

## Materials and methods

### Synthesis and characterization of liposomes

EA@lip was prepared via the “lipid film hydration and extrusion” method^35^. The detailed synthesis procedure, along with comprehensive physicochemical characterizations, is provided in the Supplementary File 1 (Sect. 1.5). These include particle size, polydispersity index (PDI), surface charge (zeta potential) measured by Dynamic Light Scattering (DLS), morphological evaluation by Transmission Electron Microscopy (TEM), phospholipid quantification via the Bartlett phosphate assay, and entrapment efficiency (EE) of EA determined spectrophotometrically at 276 nm.

#### Drug release study

Ellagic acid release from the PCL/f-MWCNT/EA@lip scaffolds was evaluated using the dialysis method in PBS buffer (pH = 7.4, 37 °C) under shaking incubation (100 rpm). Briefly, 5 × 5 cm2 PCL/f-MWCNT/EA@lip scaffolds contained approximately 6.6 mg of entrapped EA were placed in a petri dish and 75 mL of buffer was added. The release of ellagic acid was assessed during 7 days. 1 mL of buffer solution was taken out and replaced with an equal amount of fresh buffer medium at defined time-intervals (0, 0.5, 1, 2, 4, 8, 12, 24, 36, 48, 72, 96, 120, 144, and 168 h). Comparing the experimental results and calibration curve in the UV/Vis spectrophotometer measurement (UV-2600, Shimadzu, Japan) at a wavelength of 276 nm, the quantity of EA in the reserved samples was ascertained. The initial EA concentration in the buffer was approximately 88 µg/mL.

### Electrospinning and fabrication of the scaffolds

The production of nanofibers was achieved via the electrospinning technique, with further information provided in the supplementary file.

### Morphology of nanofibers

The morphology and size of the fibers were analyzed via field-emission scanning electron microscopy (FESEM), and the dispersion and morphology of the f-MWCNTs within the fibers were assessed via transmission electron microscopy (TEM). More information on these methods is available in the supplementary file.

### Characterization of the scaffolds

#### Mechanical properties

Microtensile testing was performed using a TA Plus machine (USA) equipped with a 100 N load cell at a crosshead speed of 10 mm/min. Rectangular scaffold samples (5 × 1 cm, *n* = 5) were tested in dry conditions. The initial gauge length was set to 3 cm, and the ultimate tensile strength and elongation were recorded (*n* = 5).

#### Chemical characterization

Fourier transform infrared spectroscopy (FTIR) and attenuated total reflection (ATR) were used to assess chemical composition. The f-MWCNT spectrum was acquired using the KBr disk method, while PCL-based samples were evaluated using ATR-FTIR (Thermo Nicolet Avatar 370, USA) within a scanning range of 400–4000 cm⁻¹ at 4 cm⁻¹ resolution.

#### Hydrophilicity

Hydrophilicity of the scaffolds was assessed by measuring the water contact angle (WCA) using an optical contact angle apparatus (JC2000A, China). A 3 µL drop of deionized water was placed on scaffold samples (1 × 1 cm), and images were captured at 1, 5, and 10 s (*n* = 3). ImageJ software was used for analysis.

#### Degradation and swelling behavior

To evaluate in vitro biodegradation, sterilized scaffolds (5 × 5 cm, *n* = 3) were incubated in PBS (pH 7.4, 40 mL) at 37 °C. Weight loss was measured over 56 days at weekly intervals using Eq. (1).

1 $${\text{Weight loss }}\left( \% \right){\text{ }} = {\text{ }}\left[ {\left( {{\text{W}}0 - {\text{W1}}} \right)/{\text{ W}}0} \right]{\text{ }} \times {\text{ 1}}00$$

To evaluate the water absorption and hydrophilicity of each group, the swelling ratio (SR) of the PCL and PCL/f-MWCNT scaffolds were tested (*n* = 3). After 1, 3, 5, 7, 12, 16, 20 and 24 h of immersion in PBS solution (pH = 7.4), the dry weight and the wet weight of the scaffolds were assessed to evaluate the swelling ratio (SR) of the scaffolds. The following equation was used to get the SR% (2):

2 $${\text{SR }}\left( \% \right){\text{ }} = \left[ {\left( {{\text{W2}} - {\text{W}}0} \right)/{\text{W}}0} \right]{\text{ }} \times {\text{ 1}}00$$

W0 is the weight of dried samples before soaking and W2 is the weight of soaked samples.

### 2.6. In vitro biological and biochemical assessments

The ADMSCs used in this study were originally isolated from human subcutaneous adipose tissue obtained from healthy female donors who underwent aesthetic liposuction procedures. The cells were provided by the Matin Laboratory (Ferdowsi University of Mashhad, Iran)^40^.The cells were used at passage 4 and cultured in Dulbecco’s modified Eagle’s medium (DMEM; Gibco) supplemented with 10% fetal bovine serum at 37 °C in humidified air with 5% CO_2_. After they reached confluence, the ADMSCs were passaged with 0.25% trypsin, and the cells at passage 4 were used for the following experiments.

MTT and 1,1-diphenyl-2-picrylhydrazyl (DPPH) assays were used to determine the half-maximal inhibitory concentration (IC_50_) for the cytotoxicity and radical scavenging of EA and EA@lip (*n* = 3). For both assays, the following concentrations were tested: 7.18, 14.3, 28.7, 57.5, 115, 230, and 460 µg/ml. The optimal concentration of EA was subsequently determined on the basis of the concentrations that resulted in the highest survival rates and maximum radical scavenging rates at 1, 3, and 7 days after treatment. Further information can be found in the supplementary file 1.

The MTT assay was employed to assess cell viability and proliferation by seeding cells on the samples on days 1, 3, and 7 (*n* = 3). The attachment of cells on the samples was evaluated via DAPI staining, which relies on the ability to stain intact DNA on day 3 (*n* = 3). The expansion and connection of cells with the scaffolds were examined via FE-SEM on day 3 (*n* = 3). Further information can be found in the supplementary file 1.

Biochemical parameters for measuring OS, such as LPO, reactive oxygen species (ROS), total antioxidant capacity (TAC), and total thiol molecules (TTM), were examined by exposing scaffolds containing 1 × 10^4^ ADMSCs to H_2_O_2_ for 24 h. The control groups for all the assays consisted of ADMSCs cultured on plates with or without H_2_O_2_. Prior to H_2_O_2_ treatment, the ADMSCs were cultured in the scaffolds and plates for three days (*n* = 3). More information can be found in the supplementary file 1.

### In vivo studies

#### Surgical procedure

Sixty female Wistar rats (weighing 250–300 g) were obtained from the Animal House Center of the Faculty of Medical Sciences, Mashhad University of Medical Sciences (MUMS). All rats were housed in separate cages under a 12-hour light-dark cycle, with water and food provided ad libitum. Animal welfare was monitored, and all surgical procedures were conducted in accordance with the institutional guidelines of Mashhad University of Medical Sciences (MUMS) under ethical approval (IR. MUMS. REC.1399.571). An intraperitoneal (IP) injection of a mixture containing xylazine (5 mg/kg) and ketamine (100 mg/kg) was administered to anesthetize the rats. The dorsal skin was shaved and disinfected with betadine solution. A midline incision was made, followed by T9 laminectomy using a rongeur. The exposed dura mater was incised longitudinally and retracted. Afterward, a 1 mm section of dorsal hemisection spinal tissue between the T8 and T9 vertebral levels was removed using microdissection scissors and a 22-gauge needle. Before implantation, the PCL/f-MWCNT scaffold was sterilized by UV exposure for one hour. Scaffolds with or without ADMSCs and EA@lip were implanted into the lesion site, and muscle and skin were closed separately using absorbable vicryl sutures. The dimensions of the implanted scaffolds were 6 × 3 mm, and the volume of EA@lip was 50 µl. The animals were divided randomly into six groups as follows (*n* = 10/group):

- Sham-operated group: Laminectomy without SCI (referred to as the sham group).
- SCI without treatment (referred to as SCI).
- SCI with PCL/f-MWCNTs without cells or liposomes (referred to as PC).
- Laminectomized rats receiving scaffolds containing EA@lip (referred to as PC/N). In this group, EA@lip at a concentration of 57.5 µg/ml was applied to the scaffold surface for three days. Additionally, half an hour prior to implanting the scaffold onto the spinal cord, liposomes with the same concentration and volume were also applied.
- SCI with PCL/f-MWCNT scaffolds containing ADMSCs (referred to as PC/C). For a period of three days, 10^4^ cells were seeded on the scaffold before grafting.
- SCI with PCL/f-MWCNT/EA@lip scaffolds containing ADMSCs (referred to as PC/N/C). In this group, after the scaffold was covered with liposomes, the cells were seeded onto it for three days. Then, half an hour before grafting, EA@lip at the same concentration and volume was placed on the scaffold. Further information about postoperative care and behavioral assessment can be found in supplementary file 1.

#### Assessment of OS markers

During the second week post-surgery, biochemical analyses were conducted to assess OS. This involved measuring LPO and TTM levels in the blood of the animals (*n* = 6–8). More information about these investigations can be found in the supplementary file 1.

#### Gene expression analysis

At the end of the 6-week experimental period, the remaining animals in each group were euthanized under deep anesthesia, followed by transcardial perfusion with 10% neutral buffered formalin. Real-time reverse transcription polymerase chain reaction (RT‒PCR) was used to assess the mRNA expression of particular genes (*n* = 5). Additional information regarding the procedures can be found in the supplementary file 1 (Table S1).

#### Tissue fixation and histological Preparation

After the animals were sacrificed, tissue blocks were prepared for stereological examination using cresyl violet (CV) and Luxol fast blue (LFB) staining. Quantitative evaluations involved analyzing viable neurons in the ventral and dorsal horn of the gray matter, as well as determining the axon density percentage in the dorsal column (*n* = 5). More information about these examinations can be found in the supplementary file 1.

### Statistical analysis

The experimental data were analyzed via GraphPad Prism v 9.0, and the data are presented as the means ± standard deviations (SDs). Unpaired t-tests were used for fiber diameter and mechanical strength comparisons; two-way ANOVA with Tukey’s test was applied for time-based behavioral and contact angle data; and Brown-Forsythe and Welch ANOVA with Dunnett’s T3 multiple comparisons test was used for in vivo biochemical, histochemical and gene expression data; one-way ANOVA with Tukey’s post hoc test was used for rest of in vitro assays. A significance threshold of *p* < 0.05 was used throughout.

## Results

### Morphology of the scaffolds

**Fig. 1:**
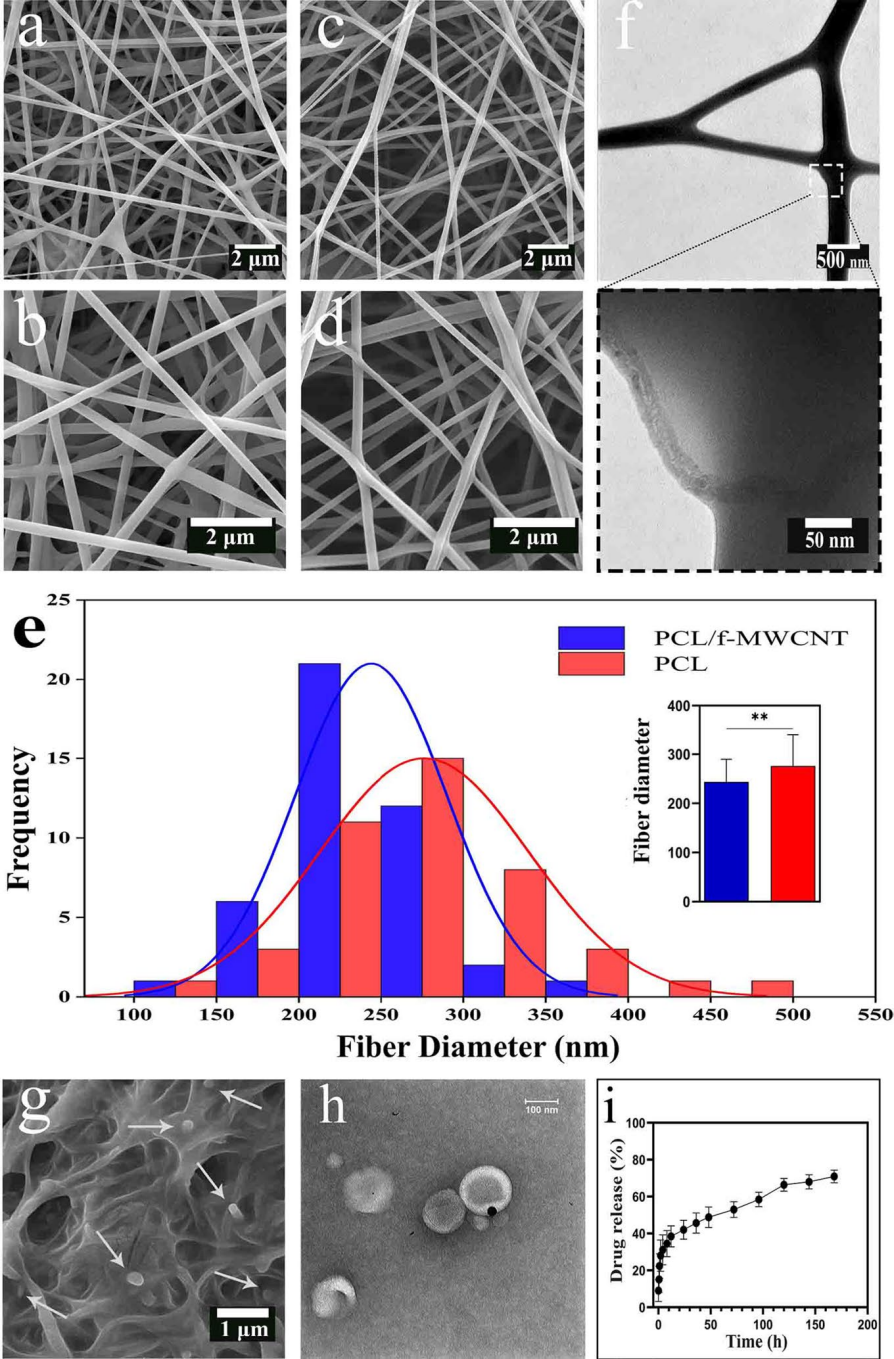
FE‒SEM micrographs of (**a** and **b**) PCLs and (**c** and d) PCL/f-MWCNTs. (**e**) Histogram showing the fiber diameter distribution for both PCL and PCL/f-MWCNT scaffolds; the inset displays the calculated average fiber diameters. (**f**) TEM image of PCL/f-MWCNT fibers. Asterisks indicate statistically significant differences (*P* < 0.01). (**g**) FE-SEM image of the PCL/f-MWCNT/EA@lip scaffold surface. (**h**) TEM images of EA-loaded liposomes, showing a nearly spherical morphology with a uniform size of approximately 100 nm. (**i**) Cumulative release profile of ellagic acid from the PCL/f-MWCNT/EA@lip scaffold over time. A rapid, burst release was observed during the first 12 h, followed by a markedly slower release phase, reaching approximately 70% by day 7.

The FE-SEM images of the PCL and PCL/f-MWCNT scaffolds (Fig. 1a-d) indicated that both electrospun scaffolds were composed of randomly oriented, bead-free and uniform nanofibers. The surface of the nanofibers was smooth, and no aggregates of MWCNTs were observed on the surface of the loaded scaffolds. This confirmed the homogeneous distribution of the f-MWCNTs within the PCL nanofibers. The mean diameter of the PCL nanofibers was 276 ± 63 nm. The addition of f-MWCNTs to PCL fibers decreased the average fiber diameter to 243 ± 45 nm (Fig. 1e). Figure 1f shows TEM images of the MWCNTs embedded within the composite fibers. It can be clearly observed that the f-MWCNTs are well embedded along the PCL nanofibers, in which the MWCNTs appear darker than the PCL matrix. Figure 1g illustrates the application of liposomes on the scaffold surface, showing a consistent and thin layer of the liposome solution on the fibers, with no signs of aggregation. TEM images of EA@lip revealed that the liposomes had approximately spherical shape with a homogeneous size of around 100 nm (Fig. 1h), consistent with their physicochemical characterization (Table S2). An in vitro release study of EA from liposome-coated scaffolds is illustrated in Fig. 1i. The release of EA from liposomes occurred rapidly and in a burst-like manner within the first 12 h. However, the release rate then decreased sharply and gradually reached 70% after 7 days.

### Inhibiting OS and enhancing the antioxidant defense system with PCL/f-MWCNT/EA@lip/ADMSC implantation following SCI

**Fig. 2:**
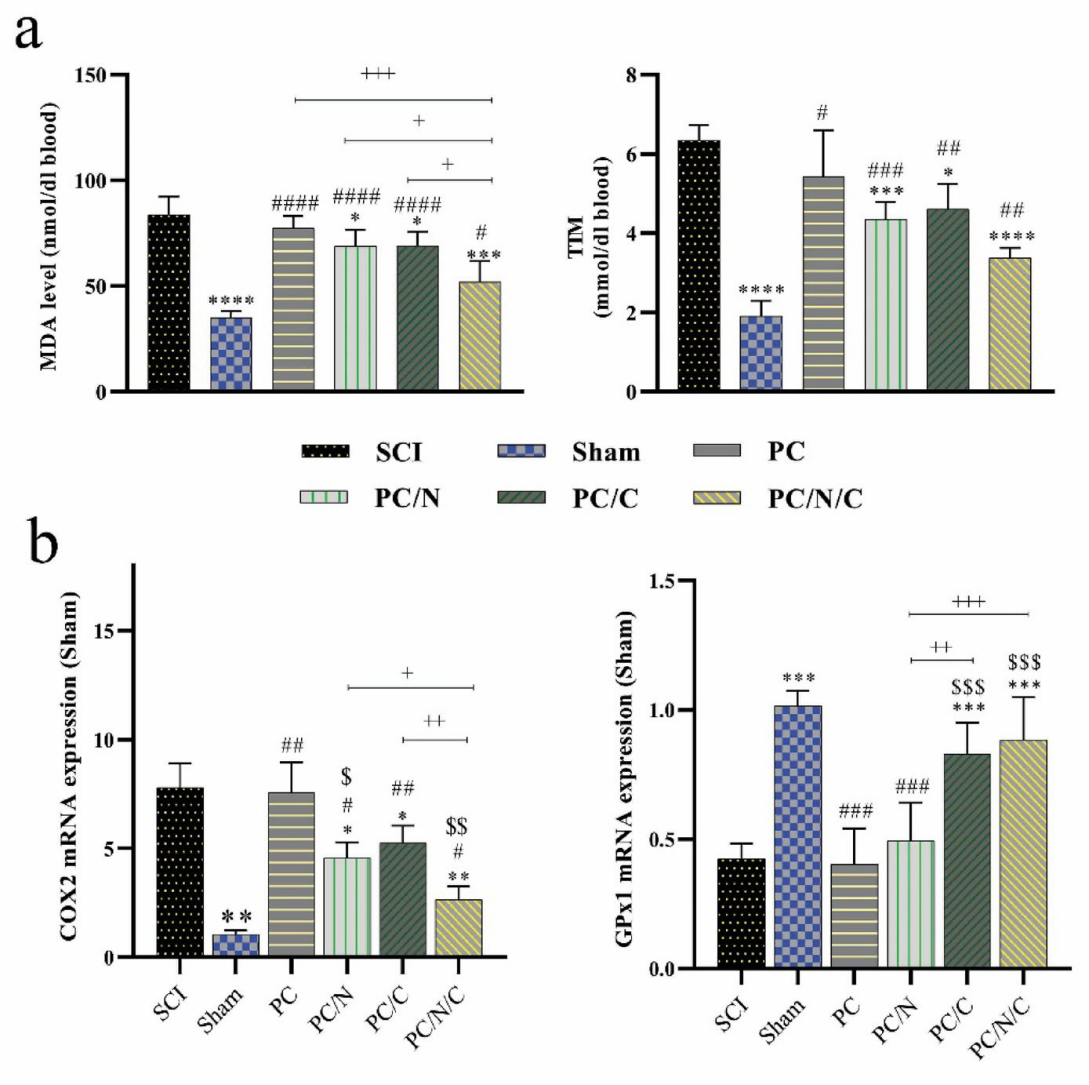
(**a**) Concentrations of malondialdehyde (MDA) and total thiol molecules (TTM) in serum samples collected two weeks after spinal cord injury (SCI). (**b**) Relative mRNA expression levels of COX2 and GPx1 in spinal cord tissue six weeks post-SCI. Data are presented as the means ± standard deviations (*n* = 5). Asterisks denote significant differences compared with the SCI group, # indicates differences compared with the Sham group, $ indicates significant differences compared with the PC group, and + indicates significant differences compared with the PC/N/C group.

SCI led to a significant increase in MDA levels and a decrease in TTM (*p* < 0.0001 and *p* < 0.0001, respectively) compared with those in the sham group after 2 weeks (Fig. 2a). Compared with the SCI group, the implantation of nanofibers alone did not alter the levels of MDA or TTM. However, the PC/N, PC/C, and PC/N/C groups showed significant reductions in MDA levels (*p* < 0.05, *p* < 0.05, and *p* < 0.001, respectively) and increases in TTM levels (*p* < 0.001, *p* < 0.05, and *p* < 0.0001, respectively) compared with the SCI group. Furthermore, the EA@lip and ADMSC-treated groups exhibited significantly lower MDA levels compared with the PC, PC/N, and PC/C groups (*p* < 0.001, *p* < 0.05, and *p* < 0.05, respectively). However, no significant differences were observed in TTM levels among the treatment groups.

To evaluate the impact of dorsal hemisection on spinal cord oxidative status, we conducted a qPCR analysis (Fig. 2b). Our findings revealed that COX2 expression was significantly higher in the SCI group than in the sham group (*p* < 0.01), whereas GPx1 expression was significantly reduced following SCI (*p* < 0.001). Interestingly, COX2 expression significantly decreased after nanofiber implantation in the PC/N, PC/C, and PC/N/C groups (*p* < 0.05, *p* < 0.05, and *p* < 0.01, respectively), whereas nanofibers alone did not induce any change in this gene. Moreover, the reduction in COX2 expression was significantly greater in the PC/N/C and PC/N groups compared with the PC group (*p* < 0.01 and *p* < 0.05, respectively). Compared with the SCI group, only the groups that contained cells presented significantly increased GPx1 expression levels, which were restored to levels comparable to those of the sham group.

### Boosting neuronal activity and histochemical integrity in Gray matter Horns with PCL/f-MWCNT/EA@lip/ADMSCs following SCI

**Fig. 3:**
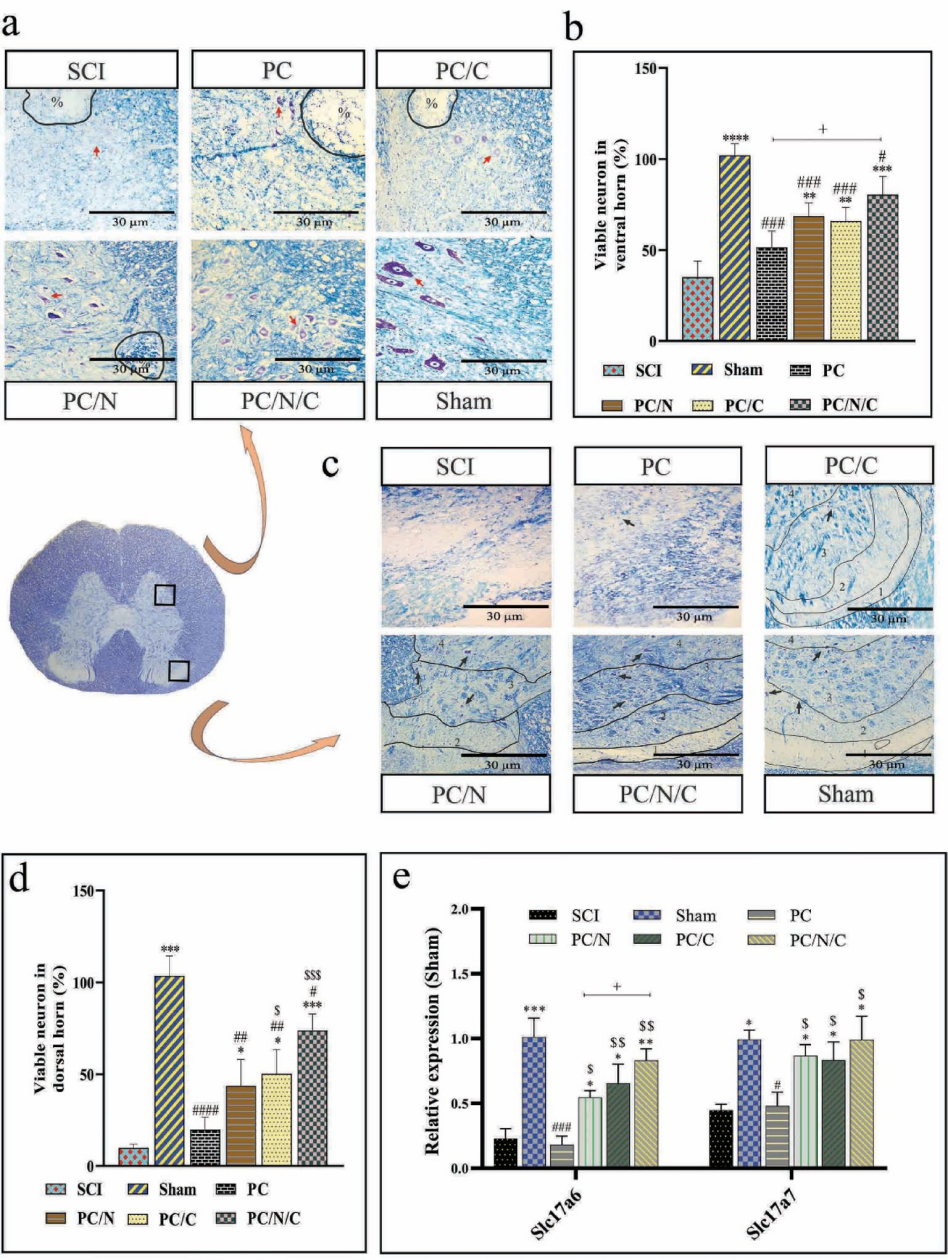
(**a**) Histological sections of the spinal cord six weeks post-SCI showing cresyl violet/Luxol fast blue staining. % indicates cyst formation and vascular rupture. The red arrow denotes a representative viable neuron in the anterior horn. (**b**) Stereological analysis of the percentage of viable neurons in the anterior horn, compared with the sham group. (**c**) Histological sections of laminae I–IV in the dorsal horn of the gray matter at six weeks post-SCI. The numbers 1–4 correspond to laminae I–IV, respectively. The black arrow marks a representative viable neuron in the posterior horn. (**d**) Stereological quantification of viable neurons in the posterior horn, compared with the sham group. (**e**) Relative mRNA expression levels of Slc17a6 and Slc17a7 in spinal cord tissue six weeks following SCI. Data are presented as the means ± standard deviations (*n* = 5).

The neuroprotective effects of the scaffolds following SCI were investigated through LFB and CV staining of the anterior and posterior horns of the spinal cord (Fig. 3a and c). The PC/N, PC/C, and PC/N/C groups exhibited an improved tissue matrix in the anterior horn and reduced cavity formation in the host tissue. Notably, the PC/N/C group showed no cyst formation in the anterior horn, in contrast to the other SCI groups. Histological analysis revealed that rats in the SCI group had fewer neurons in the anterior horns of the gray matter within a 3 mm block surrounding the injury center (Fig. 3b). Compared with SCI group, scaffold treatment significantly increased the neuronal density in the anterior horn across all treatment groups. Moreover, the number of viable neurons in the PC/N/C group was significantly higher than that in the PC group (*p* < 0.05). The histological analysis also showed extensive structural damage in laminae I–IV of the posterior horn of the gray matter in both the SCI and PC groups (Fig. 3d). In contrast, the PC/N, PC/C, and PC/N/C groups demonstrated improved matrix integrity and neuron preservation in the posterior horn. Nevertheless, matrix reconstruction in lamina I was not well restored in the PC/N group. Compared with the PC and SCI groups, the PC/N/C group exhibited the greatest degree of neuronal preservation in the posterior horn (*p* < 0.001 for both), with only a slight difference compared to the sham group (*p* < 0.05).

To assess neuronal excitability in the posterior and anterior horns, expression levels of Slc17a6 and Slc17a7 were measured (Fig. 3e). Dorsal hemisection led to a significant reduction in the expression of both genes in the SCI group compared with the sham group (*p* < 0.001 and *p* < 0.05, respectively). However, the PC/N, PC/C, and PC/N/C groups showed significantly increased expression of both genes compared with the SCI group (*p* < 0.05, *p* < 0.05, and *p* < 0.01 for Slc17a6 and *p* < 0.05, *p* < 0.05, and *p* < 0.05 for all groups for Slc17a7). Notably, the scaffold alone group did not show any significant changes in the expression of these genes compared to the SCI group.

### Revitalizing motor function after SCI: A PCL-based platform enhances myelination in posterior limb pathways

**Fig. 4:**
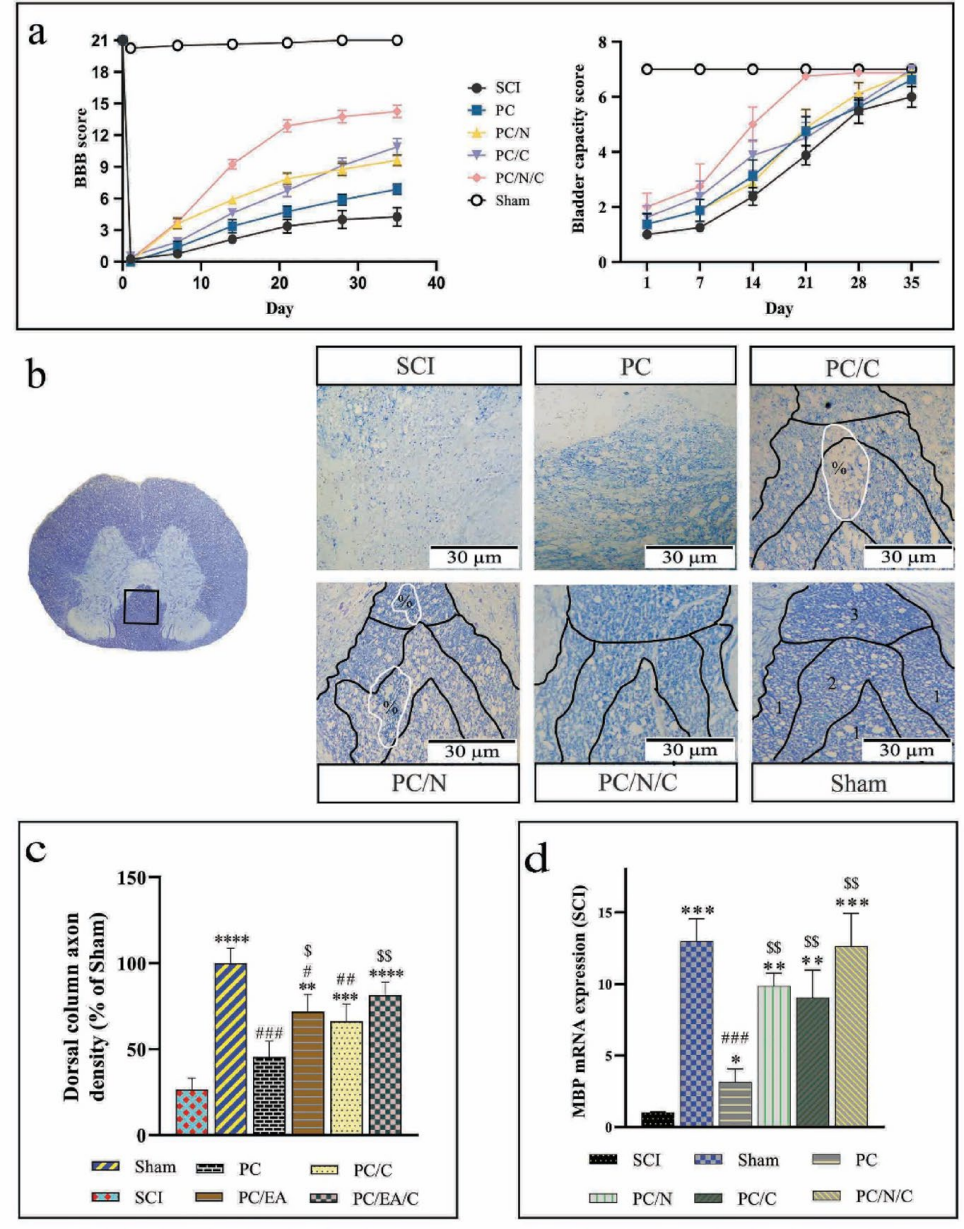
(**a**) Graphs showing the results of the Basso, Beattie, and Bresnahan (BBB) behavioral test and bladder capacity scores at six weeks post-spinal cord injury (SCI; *n* = 10). (**b**) Histological sections of the dorsal column stained with cresyl violet/Luxol fast blue. Numbers 1, 2, and 3 denote the gracile fasciculus, dorsal corticospinal tract, and postsynaptic dorsal column pathway, respectively. % indicates cyst formation and vascular rupture. (**c**) Stereological quantification of axon density in the dorsal column, expressed as a percentage relative to the sham group. (**d**) Relative mRNA expression levels of the myelin basic protein (MBP) gene in spinal cord tissue six weeks post-SCI. Data are presented as the means ± standard deviations (*n* = 5).

The hind limb motor function of the animals after SCI was assessed using the BBB behavioral test over a period of six weeks to evaluate the impact of scaffold implantation. Additionally, the test was conducted one day post-surgery to confirm successful SCI induction and associated posterior limb paralysis (Fig. 4a). The results revealed that all the animals, except those in the sham group, exhibited severe posterior limb impairment one day after surgery. During the first week, the PC/N and PC/N/C groups demonstrated faster recovery of motor function compared with the other groups. After the third week, the recovery slope for the PC/N and PC/N/C groups decreased and plateaued, whereas the performance recovery speed in the PC/C group remained unchanged. The scaffold alone group showed minimal improvements in hindlimb function. By the end of the sixth week, the PC/N/C group exhibited the greatest improvement in motor performance.

Evaluation of bladder emptying, as part of the functional recovery, revealed that all dorsal hemisection groups initially had low bladder capacity scores (Fig. 4a). However, beginning in the second week, the PC/N/C group demonstrated improved bladder function, achieving complete emptying by the third week. Other groups reached normal bladder function by the fifth week.

Photomicrographs in Fig. 4b show spinal cord sections from the postsynaptic dorsal column pathway, dorsal corticospinal tract, and gracile fasciculus, which were used to assess myelination. SCI caused severe damage to these dorsal pathways. Compared with the SCI group, treatment with nanofibers alone led to improved myelin staining and a significant increase in axon density (*p* < 0.05, Fig. 4c). However, in the PC group, the specific pathways could not be clearly distinguished. Treatment in the PC/N, PC/C, and PCNC groups preserved the dorsal myelin structure and enabled pathway differentiation. These groups also exhibited significantly increased axon density compared with the SCI group (*p* < 0.01, *p* < 0.01, and *p* < 0.0001, respectively), and the EA@lip-treated groups were significantly different from the PC group (*p* < 0.05 for PC/N and *p* < 0.01 for PC/N/C). Moreover, vacuole formation and vascular disruption were notably reduced in the PC/N/C group compared with the PC/N and PC/C groups. However, no significant differences in axon density were observed among the PC/N, PC/C, and PC/N/C groups.

Compared with the sham group, MBP mRNA expression—a key marker of myelin integrity—was significantly reduced in the SCI group (*p* < 0.001, Fig. 4d). Treatment with the PCL/f-MWCNT scaffold alone partially restored MBP transcription (*p* < 0.05 vs. SCI), but it remained significantly lower than in the sham group (*p* < 0.01). Intervention in the PC/C, PC/N, and PC/N/C groups fully restored MBP expression to levels comparable to the sham group, and were significantly different from the PC group (*p* < 0.01). Among these, the PC/N/C group demonstrated the strongest effect, showing greater differences than the SCI group (*p* < 0.001), while the PC/N and PC/C groups also showed significant but lower effects (*p* < 0.01). The utilization of the PCL/f-MWCNT/EA@lip/ADMSC platform effectively protected against tissue matrix degradation in the dorsal column and resulted in increased axonal density and MBP expression. These positive outcomes have the potential to enhance myelin formation and ultimately improve motor function in rats with SCI.

## Discussion

The increase in OS following SCI, coupled with the activation of inflammatory cells, leads to a loss of antioxidant capacity^12^. During the early phase of secondary damage, the surge in OS promotes the expression of COX2 via the NF-κB signaling pathway^41^, resulting in the destruction of basement membranes and neural structures^3^. The upregulation of COX2 is associated with the nerve tissue degradation and an increase in LPO of neuronal membranes^14^, which, in turn, produces toxic byproducts such as 4-HNE and MDA. These lipid peroxidation products initiate secondary cascades that impair cellular homeostasis and further suppress the expression of key antioxidant enzymes such as GPx and superoxide dismutase (SOD)^42^. Therefore, we detected the levels of MDA, a cytomembrane oxidation product, and TTM as important biomarkers of OS to assess whether the PCL/f-MWCNT/EA@lip/ADMSC platform ameliorated secondary cascade damage. Compared with the sham group, elevated blood MDA levels and reduced TTM levels in all experimental groups were associated with lower hindlimb motor function scores up to two weeks post-surgery. These findings suggest that dorsal hemisection SCI induces significant imbalances in mitochondrial antioxidant distribution and function in neurons, contributing to spinal cord dysfunction. Furthermore, while monitoring the animals’ behavioral performance, which stabilized by the sixth week, the SCI groups exhibited a reduction in GPx1 expression and an increase in COX2 expression.

The ability of ADMSCs to increase the expression of endogenous antioxidant enzymes (e.g., GPx1) and reduce inflammatory mediators plays a key role in restoring redox homeostasis^43^. Likewise, EA, by directly scavenging ROS and modulating signaling cascades such as Nrf2/ARE and MAPK, contributes to the suppression of oxidative and inflammatory pathways^44^. Nrf2 activation increases the transcription of antioxidant genes, while inhibiting ROS-mediated activation of COX2 and other pro-inflammatory genes^45^. These molecular mechanisms jointly underlie the therapeutic efficacy of our combinatorial platform.

In accordance with previous studies, the formation of a cellular structure depends on a an optimal balance between the strength and plasticity of the polymer matrix^46^. In the context of SCI, the use of a scaffold carrier with high mechanical strength is essential to support cellular functions and create a stable three-dimensional (3D) matrix^35^. A study conducted by Wu et al.^47^ demonstrated that f-MWCNTs with carboxylic acid groups exhibited more favorable mechanical properties than nonfunctionalized MWCNTs, primarily due to their improved dispersion within PCL fibers. The findings of our study indicate that the f-MWCNTs were uniformly dispersed along the PCL fibers, resulting in an increase in the ultimate tensile strength (UTS), Young’s modulus, and cell adhesion in the composite fibers compared with pure PCL fibers (Fig S 1–2). In accordance with Świętek et al.^48^, the cells were well spread on a high-UTS scaffold with prominent and well-developed focal adhesions.

Hierarchical fibrous morphology is highly important for synthetic neural scaffolds, as this structure mimics the architecture of the native ECM^49^. Preclinical models of SCI have demonstrated that PCL composite fiber implants are effective in preserving tissue integrity^23,50,51^. Additionally, the degree of cell adhesion depends on fiber diameter^52^. The reduced fiber diameter of the PCL/f-MWCNTs compared with that of the neat PCL scaffold is attributed to the higher conductivity of the electrospinning solution^53^, as other parameters (such as the solution composition, applied voltage, nozzle-to-collector distance, and flow rate) were kept constant. The results of the present study indicate that the obtained fiber diameter of both scaffolds fall within the acceptable range for mimicking the native ECM in the central nervous system (CNS)^54^. However, smaller fiber diameters provide a greater surface area, offer more sites for cell attachment, and exhibit superior tensile properties^55^, a condition that is clearly observed in the PCL/f-MWCNT scaffolds. In vitro studies have also shown that the incorporation of CNTs into PCL fibers enhances their electrical conductivity, which is crucial for nerve tissue regeneration^56,57^. However, in vivo results indicate that the PCL/f-MWCNT scaffold alone lacks regenerative capacity in our SCI model, suggesting that it does not significantly influence the behavior of endogenous neural stem cells or contribute to the repair of primary damage within the studied timeframe. Furthermore, our findings confirm that the modulatory effects of PCL scaffolds on SCI are primarily due to the substrate optimization for the delivery of therapeutic agents and cells, rather than any direct involvement of endogenous stem cells in the repair process^50,51^.

Based on the FE-SEM images, the scaffold alone does not provide a suitable environment for cell niche formation, growth, and adhesion. The ECM plays a crucial role in cell adhesion, proliferation, and migration^22^. Therefore, the initial step toward improving the OS environment is to enhance cell adhesion to the culture substrate^21^. The in vitro results indicate that the incorporation of EA@lip into the scaffold enhances its hydrophilicity (Fig S1) and promotes stem cell attachment (Fig S2). This finding is consistent with that of Mohammadi et al.^58^, who demonstrated that the WCA of poly-L-lactic acid scaffolds decreased from hydrophobic (130°) to completely hydrophilic (0°) upon the addition of similar liposomal components. Additionally, liposome incorporation significantly increased the OH stretching peak in the FTIR spectrum (Fig S1), which correlates with the WCA results. The molecular mechanism by which EA enhances the antioxidant system involves both direct and indirect pathways. EA can directly scavenge free radicals via hydrogen atom transfer and electron donation due to its multiple hydroxyl groups. Indirectly, it can activate the Nrf2 signaling pathway, which upregulates antioxidant genes^30,32^. Nrf2 binds to the antioxidant response element (ARE) in the promoter regions of these genes, promoting cytoprotective responses against oxidative and electrophilic stress^45^. Moreover, EA has been shown to inhibit the NF-κB pathway, thereby reducing the transcription of pro-inflammatory genes such as COX2, TNF-α, and IL-1β^59^.

The encapsulation of antioxidant drugs in liposomes improves their stability, solubility, bioavailability, and circulation time, while also reducing their toxicity and side effects^60^. Our findings demonstrate that, over time, encapsulated EA exhibits stronger antioxidant activity than its free form does (Table S3). Moreover, drug encapsulation was associated with a higher cell survival and enhanced antioxidant potential compared to the free form (Table S3). These results suggest that treatment with PCL/f-MWCNT/EA@lip increases TTM levels and reduces MDA levels. However, the MTT assay on day 1 showed no significant difference between the control group and the PCL/f-MWCNT/EA@lip scaffold group. As observed in the drug release profile, the release of EA occurred in a burst-like manner on the first day, which may explain the low cell proliferation at that time point.

Under inflammatory conditions, glial cells become activated and express elevated levels of COX2; these cells contribute to ECM degradation and damage the lipid membranes of neurons and oligodendrocytes^61^. In contrast, ADMSCs exert anti-inflammatory effects through paracrine secretion of bioactive factors and exosomal transfer of regulatory miRNAs, which suppress pro-inflammatory cytokines and enhance antioxidant defenses^62^. Our results showed that ADMSCs increased the levels of TTM and GPx1 while reducing LPO and COX2 expression in vivo. This supports the concept that MSCs can modulate redox-sensitive transcription factors to restore homeostasis^16^. Furthermore, while EA reduced LPO and MDA levels^31^ it did not significantly upregulate GPx1 expression in our model. This finding may reflect distinct but complementary mechanisms of action between ADMSCs and EA. EA primarily acts by directly scavenging radicals and inhibiting COX2, whereas ADMSCs activate endogenous enzymatic pathways through cell signaling. These data highlight that COX2 suppression is a shared target of both agents, and its inhibition represents a key strategy for attenuating secondary injury in SCI. In light of these antioxidant and anti-inflammatory findings, understanding the molecular signaling events following SCI becomes critical.

Following SCI, the exacerbation of OS not only disrupts redox homeostasis but also activates intracellular signaling cascades such as the NF-κB and MAPK pathways, which upregulate pro-inflammatory mediators including COX2, TNF-α, and IL-1β^41,63^. These molecular events intensify lipid peroxidation and glial activation, ultimately impairing neuronal survival and axonal integrity. The current study suggests that the antioxidant-rich environment provided by EA@lip, particularly when combined with ADMSCs, may interfere with these detrimental cascades by both scavenging ROS and attenuating pro-inflammatory signaling. This dual modulatory role is evidenced by the suppression of COX2 expression and restoration of GPx1 levels in vivo, indicating a shift toward an anti-inflammatory and neuroprotective phenotype. Such molecular interventions are crucial for mitigating secondary damage and facilitating remyelination and synaptic preservation, which were corroborated by histological and gene expression analyses.

Controlling extensive damage to neurons and oligodendrocytes after SCI is crucial for coordinating motor function recovery and spinal cord regeneration. Although strategies to prevent gliosis have been successful in limiting the spread of neuronal death and secondary damage^64^, the main challenge remains in preserving functional neurons and promoting remyelination in the perilesional area. The proteins Slc17a7 and Slc17a6, encoding vesicular glutamate transporters VGLUT1 and VGLUT2, respectively, are vital for excitatory neurotransmission. Both are downregulated after SCI, likely due to excitotoxicity, axonal degeneration, and oxidative stress^1,6^. Enhancing their expression may facilitate synaptic plasticity and support the maintenance of neuronal function.

Our results show that incorporation of EA@lip and ADMSCs into PCL/f-MWCNT scaffolds not only enhanced the expression of Slc17a6/7 but also increased MBP, a myelin-associated marker, suggesting the preservation of excitatory neurons and promotion of oligodendrocyte integrity. Part of the observed improvement in our SCI model following PCL/f-MWCNT/EA@lip treatment may be attributed to the pro-myelinating effects of EA. This property has previously been reported in various models of spinal cord demyelination^28,29^. Our earlier studies also showed that scaffolds containing EA@lip can enhance neuronal plasticity and electrophysiological function in regions remote from the injury site^35^. One likely mechanism is the anti-inflammatory and antioxidant action of EA, which suppresses microglial activation via COX2 downregulation, thereby mitigating chronic demyelination^5^. In line with this, our current results show that EA@lip treatment induced the expression of Slc17a6, Slc17a7, and MBP, while limiting neuronal and oligodendrocyte loss, suppressing COX2 expression, and preventing SCI-induced demyelination. Ardah et al. attributed the neuroprotective effects of EA in an Alzheimer’s disease model to its potent antioxidant properties^34^. Our findings strongly support the role of EA’s antioxidant activity in mitigating secondary injury cascades following SCI. However, we acknowledge that additional mechanisms—independent of antioxidant defense—may also contribute to the neuroprotective effects of EA.

Although MSC differentiation into neurons or glia has been demonstrated on PCL scaffolds^51^, our study did not evaluate lineage-specific differentiation. Instead, we hypothesize that the modulation of oxidative and inflammatory microenvironments by the scaffold components facilitated neuronal survival and remyelination. By suppressing ROS and LPO, and enhancing TAC and TTM, the treatment limited secondary neurodegeneration, a key pathological feature of SC^4,65^. Our findings demonstrated that the antioxidant properties of ADMSCs play a key role in mitigating both inflammation and OS, as evidenced by decreased MDA and COX2 levels. These effects contributed to increased axon density, enhanced survival of excitatory and native neurons in both the posterior and anterior horns, and improved remyelination, as indicated by elevated MBP expression. Additionally, the upregulation of GPx1 expression represents a notable advantage of the PC/C group compared with the PC/N group, highlighting the critical contribution of cell-based therapy to the observed neuroprotective effects.

Our results confirm that early burst release of EA during the acute phase (< 48 h) coincides with a critical window of ROS accumulation and inflammatory signaling activation, including TNF-α, IL-1β, and COX2 expression^10^. The initial release may have counteracted these pathways, reducing tissue damage. Meanwhile, the sustained release profile observed during the subacute and early chronic phases likely prevented delayed neuroinflammation and reactivation of glial responses, as described in chronic SCI models^15^. This pharmacokinetic profile enhanced axonal density, neuronal survival in both horns, and MBP expression, resulting in superior outcomes in the PC/N/C group.

Despite these promising results, a limitation of our study was that mechanical properties were only tested under dry conditions. In vivo, hydration significantly alters scaffold compliance and mechanical behavior. Future studies should consider evaluating the mechanical performance of scaffolds under physiological wet conditions to provide a more accurate representation of their functional integrity post-implantation. Lastly, while the in vitro degradation profile suggested slow hydrolysis, in vivo results indicated complete scaffold degradation. This might be due to enhanced macrophage infiltration, hydrophilic modification by f-MWCNTs, and enzymatic degradation within the injured tissue. However, despite these positive features, full functional recovery was not achieved, indicating the need for advanced bioactive materials with tailored degradation, controlled spatiotemporal release of neuroprotective agents, and multi-modal therapeutic potential. The synergistic action of antioxidant-loaded nanofibers and stem cells contributes to the preservation of spinal architecture, reduction of oxidative damage, suppression of COX2-driven inflammation, and enhancement of remyelination, offering a promising foundation for future translational approaches in SCI repair.

## Conclusion

The objective of this study was to develop a biomimetic scaffold incorporating ADMSCs and enabling the controlled release of EA to improve tissue structure and mitigate oxidative cascade damage in SCI. The EA@lip exhibited favorable particle size and zeta potential. Moreover, the encapsulation of EA enhanced its radical scavenging capacity (increased IC₅₀) and significantly reduced cytotoxicity. We successfully fabricated PCL/f-MWCNT nanofibrous scaffolds with interconnected, bead-free networks via electrospinning. The integration of f-MWCNTs resulted in a decrease in fiber diameter, while their alignment along the nanofibers contributed to enhanced mechanical strength, swelling ratio, and accelerated degradation of the PCL matrix. The hydrophilicity of the scaffolds was markedly improved by the incorporation of EA@lip. Among all tested formulations, the PCL/f-MWCNT/EA@lip scaffold exhibited the highest levels of ADMSC attachment and proliferation, as demonstrated by MTT assay, DAPI staining, and FE-SEM analysis. In vivo, this platform effectively regulated oxidative stress by reducing LPO and COX2 levels and enhancing the antioxidant defense system, as evidenced by increased TTM and GPx1 levels. Improvement in behavioral performance was supported by histological and cellular analyses, along with upregulated MBP expression. Furthermore, the increased expression of Slc17a6 and Slc17a7 contributed to the restoration of neuronal excitability in the posterior and anterior horns of the spinal cord. The scaffold’s high hydrophilicity, mechanical robustness, biodegradation profile, and excellent biological performance—including antioxidant and anti-inflammatory properties, and support for stem cell proliferation, adhesion, and differentiation—collectively suggest that this platform is a promising candidate for future applications in neural tissue engineering.

## Supplementary Information

Below is the link to the electronic supplementary material.

Supplementary Material 1

Supplementary Material 2

## Data Availability

The raw data supporting the findings and conclusions of this study are available from the corresponding author upon reasonable request.
