# Supplementary material for: Liposomal ellagic acid enhances the regenerative potential of ADMSC-laden nanofibrous PCL scaffolds in a rat model of spinal cord injury
Source: Sci Rep. 2025 Aug 18;15:30202. doi: 10.1038/s41598-025-15789-w (PMC12361420; doi:10.1038/s41598-025-15789-w)
Supplement: Supplementary file 1 — Supplementary Material 1 [file 41598_2025_15789_MOESM1_ESM.docx]

**Supplementary File 1**

**Liposomal ellagic acid enhances the regenerative potential of ADMSC-laden nanofibrous PCL scaffolds in a rat model of spinal cord injury**

**1. Material and methods**

**1.1. Materials**

Polycaprolactone (PCL) with an average molecular weight (Mn) of 80,000, chloroform (≥99%), Luxol fast blue (LFB), cresyl violet (CV), and methanol (≥99.5%) were purchased from Sigma-Aldrich. Multi-walled carbon nanotubes (MWCNTs) with a purity of >95% and an outer diameter of 20–30 nm were obtained from US Research Nanomaterials, Inc. (USA). Ellagic acid (EA) was acquired from Sami Labs Limited (Bengaluru, Karnataka, India). Hydrogenated soy phosphatidylcholine (HSPC), cholesterol (Chol), and distearoyl-glycero-3-phosphoethanolamine-methoxy polyethylene glycol-2000 (mPEG2000-DSPE) were obtained from Avanti Polar Lipids (Alabaster, USA). 3-(4,5-Dimethylthiazol-2-yl)-2,5-diphenyltetrazolium bromide (MTT), DMSO, phosphate-buffered saline (PBS), thiobarbituric acid (TBA), trichloroacetic acid, tri(2-pyridyl)-s-triazine (TPTZ), hydrochloric acid (HCl), n-butanol, ferric chloride hexahydrate (FeCl₃·6H₂O), ethanol, and chloroform were purchased from Merck (Germany). Dulbecco’s Modified Eagle’s Medium (DMEM), fetal bovine serum (FBS), 0.25% trypsin-EDTA, and penicillin/streptomycin were supplied by Gibco (Thermo Fisher Scientific, USA). Dialysis membranes with a molecular weight cut-off of 12–14 kDa were purchased from Spectrum Laboratories Inc. 4′,6-diamidino-2-phenylindole (DAPI), paraformaldehyde (PFA), glutaraldehyde, and Triton X-100 were obtained from Sigma-Aldrich (St. Louis, Missouri, USA). Adipose-derived mesenchymal stem cells (ADMSCs) were isolated from human adipose tissue and provided by the Matin Laboratory, Faculty of Sciences, Ferdowsi University of Mashhad, Iran.

**1.2. Synthesis of liposome**

The manufacturing conditions for ellagic acid-loaded liposomes (EA@lip) were established based on our previous study (1). A@lip was prepared using the lipid film hydration and extrusion method. Briefly, lipids including HSPC, Cholesterol, and mPEG2000-DSPE at a molar ratio of 55:40:5 were dissolved in chloroform. The organic solvent was then removed using a rotary evaporator to form a thin lipid film, which was subsequently freeze-dried to ensure complete solvent removal. The lipid film was hydrated in a 5% dextrose solution (pH 7.4) and vortexed at 65 °C to generate multilamellar vesicles. For drug loading, ellagic acid (2 mg/mL) was added to the hydration medium and incubated for 1 hour at 60 °C. Unencapsulated EA was removed via centrifugation (5 min, 1500 rpm) followed by dialysis (MWCO 12–14 kDa) against dextrose solution. Finally, the EA@lip formulation was sterilized using a 0.2 µm syringe filter.

**1.3. Electrospinning and fabrication of scaffolds**

Multi-walled carbon nanotubes (MWCNTs) were functionalized with carboxyl (COOH) groups by refluxing in a 1:1 mixture of concentrated H₂SO₄ and HNO₃. Initially, the mixture was sonicated for 30 minutes to disperse agglomerated nanotubes, followed by reflux at 120 °C for 3 hours. The resulting product was washed several times with distilled water to remove residual acid, and the purified functionalized MWCNTs (f-MWCNTs) were dried in an oven at 50 °C.

To prepare the electrospinning solution, polycaprolactone (PCL) was dissolved in a chloroform:methanol mixture (3:1 v/v) at a polymer concentration of 14% w/w and stirred overnight to obtain a homogeneous solution. For the preparation of the PCL/f-MWCNT electrospinning solution, f-MWCNTs were first ultrasonicated in methanol for 30 minutes and stirred for an additional 2 hours. The dispersed f-MWCNTs were then added to the PCL solution. Based on previous reports demonstrating superior performance in neural tissue engineering, the f-MWCNT content was fixed at 3 wt% relative to the polymer mass (2,3). Electrospinning was performed using a custom machine (Fanavaran Nano-Meghyas Co., Iran). The solution was loaded into a plastic syringe fitted with a metal needle. The needle-to-collector distance was set at 15 cm, with a flow rate of 1 mL/h and a rotating drum speed of 800 rpm. The collector drum, covered with aluminum foil, was grounded, and a high voltage of 30 kV was applied to the spinneret to initiate fiber formation.

**1.4. Morphology of scaffolds**

The morphology of the scaffolds was examined using a field emission scanning electron microscope (FESEM, Tescan Mira3 LMU, Czech Republic) operated at an accelerating voltage of 10 kV. Prior to imaging, samples were mounted on metal stubs using conductive double-sided carbon tape and sputter-coated with a thin layer of gold to enhance conductivity. The average fiber diameter was quantified using ImageJ software, with measurements taken from at least 40 randomly selected fibers per sample. To visualize the internal dispersion of f-MWCNTs within the nanofibers, an ultra-thin layer of the electrospun scaffold was deposited directly onto copper (Cu) grids. Transmission electron microscopy (TEM) was performed using a Hitachi H7100 microscope operating at an accelerating voltage of 200 kV.

**1.5. Characterization of liposomes**

The particle size, polydispersity index (PDI), and zeta potential (surface charge) of EA-loaded liposomes (EA@lip) were determined using a dynamic light scattering (DLS) instrument (Nano-ZS, Malvern Instruments, UK). The morphological characteristics of the liposomes were observed by transmission electron microscopy (TEM; Jena, Germany) operated at an accelerating voltage of 80 kV. The total phospholipid content was quantified using the Bartlett phosphate assay. The entrapment efficiency (EE) of ellagic acid was calculated based on the difference between the total amount of EA initially added (TE) and the amount of non-entrapped (free) EA, determined after separation by centrifugation and dialysis. The concentration of EA was measured using a UV–Visible spectrophotometer (UV-2600, Shimadzu, Japan) at a wavelength of 276 nm. The entrapment efficiency was calculated according to the following equation:

EE (%) = (mg of EnE /mg of TE) × 100 (1)

**1.6. In vitro biological and biochemical assessments**

ADMSCs were seeded into 24- and 96-well culture plates at a density of 1 × 10⁴ cells per well and incubated in a humidified atmosphere at 37 °C with 5% CO₂ for 2 hours. Cells were then pretreated for 1, 3, and 7 days with various concentrations of free ellagic acid (EA) and liposome-encapsulated EA (EA@lip) at 7.18, 14.3, 28.7, 57.5, 115, 230, and 460 µg/mL. The half-maximal inhibitory concentration (IC₅₀) of each formulation was determined using the MTT assay. The antioxidant activity of EA and EA@lip was assessed by the 1,1-diphenyl-2-picrylhydrazyl (DPPH) radical scavenging assay, as previously described (8). Briefly, EA and EA@lip samples were diluted to the same concentration range (7.18–460 µg/mL) and added to Tris–HCl buffer (100 mM, pH 7.0) containing 250 µM DPPH dissolved in ethanol. Samples were incubated in the dark at room temperature for 1, 3, and 7 days. The absorbance was measured at 517 nm using a spectrophotometer. Results were expressed as percent radical scavenging and IC₅₀ values. The optimal concentration of EA was determined as the dose that provided both maximum cell viability and maximum radical scavenging activity at all three time points. The percentage of DPPH radical scavenging was calculated using the following formula:

$\%Scavenging=$($\frac{A_{control}- A_{sample}}{A_{control}}$) $\times100$

Where A_control​_ is the absorbance of the DPPH solution without sample and A_sample_​ is the absorbance in the presence of EA or EA@lip.

The IC_50_ value (the concentration of compound required to scavenge 50% of DPPH radicals) was calculated by plotting % scavenging against the logarithm of concentration and fitting a nonlinear regression curve using GraphPad Prism v9.0.

Cell viability and proliferation on the scaffolds were evaluated using the MTT assay, with cells cultured on scaffolds and tissue culture plates (serving as the control). ADMSCs were cultured for 1, 3, and 7 days, after which 20 μL of MTT reagent was added to each well. The plates were incubated for 4 hours at 37 °C. Following incubation, dimethyl sulfoxide (DMSO) was added to each well to dissolve the formazan crystals, and the plates were placed on a shaker for 30 minutes to ensure complete dissolution. Absorbance was measured at 570 nm using a microplate reader spectrophotometer (SPECTROstar Nano, BMG LABTECH, Germany). For each scaffold, three wells without cells were included, and the absorbance values from cell-seeded samples were corrected by subtracting the average background absorbance from the corresponding scaffold-only wells. To assess cellular attachment, DAPI staining was performed three days post-seeding. The culture medium was removed, and the scaffolds were gently rinsed with phosphate-buffered saline (PBS). Cells were fixed in 4% paraformaldehyde (PFA) for 30 minutes at 4 °C, permeabilized with 0.2% Triton X-100 in PBS for 5 minutes, and stained with DAPI solution for 10 minutes. After washing with PBS to remove excess stain, samples were visualized under a fluorescence microscope (Olympus, Japan) equipped with 360 nm excitation and 460 nm emission filters. The morphology of ADMSCs cultured on different scaffolds was evaluated by field emission scanning electron microscopy (FE-SEM) after three days. Samples were fixed in 2.5% (v/v) glutaraldehyde at 4 °C for 3 hours, rinsed with PBS, and dehydrated through a graded ethanol series (30%, 70%, 90%, 96%, and 100% v/v, 10 minutes each). The dehydrated samples were placed in a desiccator, sputter-coated with a thin layer of gold, and imaged using FE-SEM.

Lipid peroxidation (LPO) was assessed using the thiobarbituric acid (TBA) method. Briefly, homogenized scaffold samples were mixed with 900 μL of trichloroacetic acid and centrifuged at 1500 rpm for 25 minutes. Subsequently, 160 μL of TBA (1% w/v) was added to the supernatant, and the mixture was incubated in a boiling water bath for 15 minutes. After cooling, 380 μL of n-butanol was added, and the absorbance of the resulting solution was measured at 532 nm. Reactive oxygen species (ROS) were quantified using 2′,7′-dichlorofluorescein diacetate (DCFDA), a cell-permeable fluorogenic dye. Upon cellular uptake, DCFDA was deacetylated by intracellular esterases and subsequently oxidized by ROS to form fluorescent dichlorofluorescein (DCF). Fluorescence intensity was measured using a microplate fluorimeter with excitation at 488 nm and emission at 525 nm. Total antioxidant capacity (TAC) was determined using the ferric reducing antioxidant power (FRAP) assay, which is based on the reduction of the Fe^3+-tripyridyltriazine (TPTZ) complex to the ferrous form (Fe^2+-TPTZ), producing an intense blue color at low pH. The FRAP reagent was freshly prepared by mixing 18 mmol/L FeCl₃, 280 mmol/L acetate buffer (pH 3.6), and 12 mL of 10 mmol/L TPTZ dissolved in 50 mmol/L HCl. Diluted samples were added to the FRAP reagent, and absorbance was measured at 593 nm. The level of total thiol molecules (TTM) was measured to assess antioxidant thiol capacity. Samples were mixed with Tris-EDTA buffer followed by the addition of 5,5′-dithiobis-(2-nitrobenzoic acid) (DTNB). After incubation at 25 °C for 20 minutes, the mixture was centrifuged at 3000 rpm for 12 minutes. The absorbance of the supernatant was measured at 412 nm using a microplate reader.

**1.7. In vivo studies**

**1.7.1.** **Post-surgery care**

Following surgery, all rats received gentamicin (6 mg/kg, intraperitoneally) once daily for seven consecutive days to prevent infection. Analgesia was provided with buprenorphine hydrochloride (1 mg/kg, subcutaneously) administered twice daily for three days. Animals were housed at a controlled temperature of 27 °C until full recovery from anesthesia. To maintain hydration, a subcutaneous injection of 4 mL of dextrose solution (5%) was administered immediately post-surgery, followed by 2.5 mL daily for three subsequent days. During the survival period, rats were housed in pairs. Bladders were manually expressed twice daily using the Credé maneuver until spontaneous reflexive voiding resumed. Animals were monitored closely for signs of distress and were cleaned and dried regularly to maintain hygiene and minimize the risk of infection.

**1.7.2. Behavioral assessment**

**1.7.2.1. Hind limb motor function assessment**

One day prior to surgery, baseline locomotor activity was evaluated for all rats using the 21-point Basso, Beattie, and Bresnahan (BBB) Locomotor Rating Scale (9), where a score of 0 indicates complete hindlimb paralysis and a score of 21 denotes normal locomotion. Following spinal cord injury (SCI), hindlimb motor function was reassessed using the same scale. Animals were placed individually in a 1-meter diameter open-field arena and observed for 4 minutes. Assessments were conducted one day post-surgery to confirm injury induction and subsequently on a weekly basis for six weeks. Scoring was performed by two independent observers blinded to the experimental group allocations to ensure unbiased evaluation.

**1.7.2.2. Bladder Function Assessment**

Following dorsal hemisection SCI, urinary retention was managed by manual bladder expression up to three times per day until the reappearance of reflexive voiding. Functional recovery of the bladder was monitored over a 6-week period by recording the average volume of urine manually expressed during routine care. A “bladder functionality score” was assigned based on bladder size assessed through palpation, with a score of 1 indicating a large, distended bladder and a score of 7 representing normal bladder emptying (10). Scoring was performed on days 1, 7, 14, 21, 28, and 35 post-surgery, and the mean score was calculated for each animal to evaluate the progression of functional recovery.

**1.7.3.** **Assessment of** **OS markers**

**1.7.3.1. Determination of the MDA level**

Malondialdehyde (MDA), a byproduct of lipid peroxidation (LPO), forms a red-colored complex upon reaction with thiobarbituric acid (TBA). Two weeks post-injury, 1 mL of whole blood was collected and mixed with 2 mL of a reagent solution containing 15 g/L trichloroacetic acid (TCA), 2 mL of hydrochloric acid (HCl), and 0.375 g of TBA in a centrifuge tube. The mixture was incubated in a boiling water bath for 50 minutes, then allowed to cool. Samples were centrifuged at 1000 rpm for 10 minutes, and the absorbance of the supernatant was measured at 535 nm. MDA concentration was calculated using the following equation:

$$C (M)=\frac{A}{1.56\times105}$$

**1.7.3.2. Total thiol content measurement**

Total sulfhydryl (–SH) groups were quantified using 5,5′-dithiobis-(2-nitrobenzoic acid) (DTNB), which reacts with thiol groups to produce a yellow-colored complex measurable at 412 nm. For each sample, 1 mL of Tris-EDTA buffer was added to 50 μL of blood, and the absorbance was measured at 412 nm against a buffer blank (A₁). Subsequently, 20 μL of DTNB reagent (10 mM in methanol) was added to the mixture, incubated for 10 minutes at room temperature, and the absorbance was recorded again (A₂). The absorbance of the DTNB reagent alone was also measured as a blank (B). The total thiol concentration (in mM) was calculated using the following equation:

$$Total thiol concentration (mM)=\frac{(A2-A1-B)\times1.07}{0.05\times13.6}$$

**1.7.4. Gene expression analysis**

To quantify mRNA expression levels of selected genes, real-time reverse transcription polymerase chain reaction (qRT-PCR) was performed. Animals (n=5 per group) were euthanized using the same anesthetic protocol as during surgery, and a 1 cm segment of the spinal cord at the injury epicenter was harvested, snap-frozen in liquid nitrogen, and stored at –80°C until further use. Total RNA was extracted using a High Pure RNA Isolation Kit (Roche, Germany) following the manufacturer’s instructions. Complementary DNA (cDNA) was synthesized from 45 ng of RNA using the TaqMan Reverse Transcription Kit (Applied Biosystems, USA). qRT-PCR was carried out on a StepOne™ Real-Time PCR System (Applied Biosystems, USA) using gene-specific primers obtained from previously published studies. GAPDH was used as the internal reference gene. Relative expression levels were calculated using the comparative 2^−ΔΔCt method. The PCR cycling conditions were: initial pre-incubation at 95°C for 10 seconds and 42°C for 5 minutes, followed by amplification consisting of 40 cycles at 95°C for 10 seconds, 57°C for 1 minute, and 72°C for 10 seconds. Melting curve analysis was performed at 95°C for 5 seconds and 60°C for 1 minute. Primer sequences used for each target gene are listed in Table S1.

Table S1. Primer sequences used for quantitative real-time PCR (qRT-PCR) analysis of gene expression in spinal cord tissue.

| **Gene** | **Primer** | **Ref**. |
| --- | --- | --- |
| GPx1 | \| Forward: 5’-GGAGAATGGCAAGAATGAAGA-3’ \| \| --- \| \| Reverse: 5’-CGCAGGAAGGTAAAGAG-3’ \| | (11) |
| COX2 | \| Forward: 5’-GAACAACATTCCCTTCCTTCG-3’ \| \| --- \| \| Reverse: 5’-GAAGTTCCTTATTTCCTTTCACACC-3’ \| | (12) |
| Slc17a6 | \| Forward: 5’-GCAGTGGGATTCAGTGGATT-3’ \| \| --- \| \| Reverse: 5’-CCATTCTTCACGGGACTTGT-3’ \| | (13) |
| Slc17a7 | \| Forward: 5’-CCTTAGAACGGAGTCGGCTG-3’ \| \| --- \| \| Reverse: 5’-AAGATCCCGAAGCTGCCATA-3’ \| | (14) |
| MBP | \| Forward: 5’-AGTCGCAGAGGACCCAAGAT-3’ \| \| --- \| \| Reverse: 5’-GACAGGCCTCTCCCCTTTC-3’ \| | (15) |
| GAPDH | \| Forward: 5’-ACATCAAATGGGGTGATGCT-3’ \| \| --- \| \| Reverse: 5’-GTGGTTCACACCCATCACAA-3’ \| | (14) |

**1.7.5.** **Tissue Fixation and Histological Preparation**

The spinal cords were harvested, and paraffin blocks containing the lesion epicenter and adjacent tissue (+1 mm from the lesion center) were prepared. Serial longitudinal sections (5 μm thick) were obtained using a rotary microtome (MR 3000, Histo-Line, Italy), with an interslice interval of 25 μm (H). Every 8th section was selected for analysis, yielding approximately 9 sections per animal (n = 5). To visualize neuronal and myelin structures, sections underwent histological differentiation using cresyl violet (CV) and Luxol fast blue (LFB) staining. Briefly, paraffin sections were deparaffinized and rehydrated, followed by immersion in LFB solution at 60°C for 18 hours. This was followed by counterstaining with CV for 10 minutes at 40°C. Stained sections were mounted and examined under a light microscope equipped with a digital camera (BX51, Olympus, Japan) using a 20× objective. Stereological analyses were performed on the anterior horn (laminae VII–X), posterior horn (laminae I–V), and dorsal column regions of the spinal cord (including the postsynaptic dorsal column pathway, dorsolateral fasciculus, dorsal corticospinal tract, and gracile fasciculus). Two fields per region were randomly selected from each slide. To estimate neuronal density, each image was superimposed with a 2×2 grid, and six random sampling squares (100 × 100 μm²) were selected for counting viable neurons, which were identified by their morphology (intact cytoplasm, clear nucleus, and nucleolus). Neuron counts were performed using Mosaic Software (USA), and neuronal density (ND) was calculated using the following equation:

$$ND = \frac{Sum of neurons counted in a sample}{Counting frame area} \times GS \times H$$

The density of viable neurons in the anterior (ventral) and posterior (dorsal) horns of the spinal cord was quantified for each experimental group and expressed as a percentage relative to the sham group, which served as the baseline reference.

To evaluate remyelination, the axonal density in the dorsal column was calculated by determining the proportion of the area occupied by axons relative to the total area of the dorsal column. For this analysis, high-resolution histological images were randomly selected and processed using ImageJ software. First, the total dorsal column area was delineated and measured. Then, an image mask was applied to exclude cystic or empty regions, allowing for the selective measurement of axonal regions within the column. The axonal area was normalized to the total dorsal column area, and results were expressed as percentages relative to the sham group, which represented the uninjured baseline.

**References**

1. Abroumand Gholami A, Gheybi F, Molavi AM, Tahmasebi F, Papi A, Babaloo H. Effect of polycaprolactone/carbon nanotube scaffold implantation along with liposomal ellagic acid in hippocampal synaptogenesis after spinal cord injury. Nanomedicine J [Internet]. 2023;10(3):197–209. Available from: https://nmj.mums.ac.ir/article_22560.html

2. Gattazzo F, De Maria C, Whulanza Y, Taverni G, Ahluwalia A, Vozzi G. Realisation and characterization of conductive hollow fibers for neuronal tissue engineering. J Biomed Mater Res Part B Appl Biomater. 2015;103(5):1107–19.

3. Yousefi Talouki P, Tehrani P, Shojaei S. The Relationship between Thermomechanical Properties with Morphology in PCL/PHBV/MWCNT Biodegradable Nanocomposites with Application in Neural Tissue Engineering. Razi J Med Sci. 2021;27(11):25–38.

4. Bartlett GR. Phosphorus assay in column chromatography. J Biol chem. 1959;234(3):466–8.

5. Chi Y, Yin X, Sun K, Feng S, Liu J, Chen D, et al. Redox-sensitive and hyaluronic acid functionalized liposomes for cytoplasmic drug delivery to osteosarcoma in animal models. J Control Release [Internet]. 2017;261:113–25. Available from: https://www.sciencedirect.com/science/article/pii/S0168365917306922

6. Díaz E, Sandonis I, Valle MB. In vitro degradation of poly (caprolactone)/nHA composites. J Nanomater. 2014;2014:185.

7. Li X, Wang C, Yang S, Liu P, Zhang B. Electrospun PCL/mupirocin and chitosan/lidocaine hydrochloride multifunctional double layer nanofibrous scaffolds for wound dressing applications. Int J Nanomedicine. 2018;5287–99.

8. Yang Z, Zheng Y, Cao S. Effect of high oxygen atmosphere storage on quality, antioxidant enzymes, and DPPH-radical scavenging activity of Chinese bayberry fruit. J Agric Food Chem. 2009;57(1):176–81.

9. Basso DM, Beattie MS, Bresnahan JC. A sensitive and reliable locomotor rating scale for open field testing in rats. J Neurotrauma. 1995;12(1):1–21.

10. Romanelli P, Bieler L, Heimel P, Škokić S, Jakubecova D, Kreutzer C, et al. Enhancing functional recovery through intralesional application of extracellular vesicles in a rat model of traumatic spinal cord injury. Front Cell Neurosci. 2022;15:795008.

11. Ebrahimpour S, Shahidi SB, Abbasi M, Tavakoli Z, Esmaeili A. Quercetin-conjugated superparamagnetic iron oxide nanoparticles (QCSPIONs) increases Nrf2 expression via miR-27a mediation to prevent memory dysfunction in diabetic rats. Sci Rep [Internet]. 2020;10(1):15957. Available from: https://doi.org/10.1038/s41598-020-71971-2

12. Adachi K, Yimin Y, Satake K, Matsuyama Y, Ishiguro N, Sawada M, et al. Localization of cyclooxygenase-2 induced following traumatic spinal cord injury. Neurosci Res [Internet]. 2005;51(1):73–80. Available from: https://www.sciencedirect.com/science/article/pii/S0168010204002573

13. Homma T, Sakakibara M, Yamada S, Kinoshita M, Iwata K, Tomikawa J, et al. Significance of neonatal testicular sex steroids to defeminize anteroventral periventricular kisspeptin neurons and the GnRH/LH surge system in male rats. Biol Reprod. 2009;81(6):1216–25.

14. Abuaish S, Al-Otaibi NM, Abujamel TS, Alzahrani SA, Alotaibi SM, AlShawakir YA, et al. Fecal transplant and Bifidobacterium treatments modulate gut Clostridium bacteria and rescue social impairment and hippocampal BDNF expression in a rodent model of autism. Brain Sci. 2021;11(8):1038.

15. Labombarda F, González SL, Lima A, Roig P, Guennoun R, Schumacher M, et al. Effects of progesterone on oligodendrocyte progenitors, oligodendrocyte transcription factors, and myelin proteins following spinal cord injury. Glia [Internet]. 2009 Jun 1;57(8):884–97. Available from: https://doi.org/10.1002/glia.20814
