# Supplementary material for: Liposomal ellagic acid enhances the regenerative potential of ADMSC-laden nanofibrous PCL scaffolds in a rat model of spinal cord injury
Source: Sci Rep. 2025 Aug 18;15:30202. doi: 10.1038/s41598-025-15789-w (PMC12361420; doi:10.1038/s41598-025-15789-w)
Supplement: Supplementary file 2 — Supplementary Material 2 [file 41598_2025_15789_MOESM2_ESM.docx]

**Supplementary File 2**

**Liposomal ellagic acid enhances the regenerative potential of ADMSC-laden nanofibrous PCL scaffolds in a rat model of spinal cord injury**

**3. Results**

**3.1. Physicochemical characterization of EA@lip**

The physicochemical characteristics of EA@liposomes, including particle size (nm), surface charge (mV), polydispersity index (PDI), entrapment efficiency (EE%), and lipid concentration, are summarized in Table S1. The average sizes of blank liposomes (Lip) and ellagic acid-loaded liposomes (EA@lip) were 119 ± 5.36 nm and 129.4 ± 2.05 nm, respectively, indicating that ellagic acid did not significantly affect liposome size. All formulations exhibited negative zeta potentials, and the PDI values were below 0.3, indicating good size uniformity. The entrapment efficiency of EA@lip was measured at 33%. Liposomes were prepared in a 100 mM lipid concentration using dextrose-based hydration buffers (Table S2). Figure 1g presents FE-SEM images of the EA@lip layer coated on the surface of the PCL/f-MWCNT scaffold. Figure 1h shows a TEM image of the EA@lip, revealing nearly spherical vesicles with a homogeneous diameter of approximately 100 nm and uniform morphology, which is consistent with the dynamic light scattering (DLS) results reported in Table S2.

Table S2. Physicochemical characteristics of liposomes

|  | Lipid composition | Molar ratio | Particle size (nm ± SD) | PDI ± SD | Zeta-potential (mV) ± SD | EE% | Lipid concentration (mM) |
| --- | --- | --- | --- | --- | --- | --- | --- |
| Lip | HSPC/Chol/ mPEG2000-DSPE | 55:40:5 | 119 ± 5.36 | 0.21 ± 0.11 | -6.79 ± 3.76 | -- | 100 |
| EA@lip | HSPC/Chol/ mPEG2000-DSPE | 55:40:5 | 129.4 ± 2.05 | 0.29 ± 0.07 | −7.92 ± 5.39 | 33 | 100 |

Data represented as Mean ± standard deviation.

**3.2. Enhancement of scaffold's physical and chemical properties by f-MWCNT**

Fig S1a shows the stress–strain curves and corresponding mechanical parameters, including ultimate tensile strength (UTS), strain at break, and Young’s modulus for the PCL and PCL/f-MWCNT scaffolds. For the PCL scaffold, the UTS, elongation at break, and Young’s modulus were measured as 1.5 MPa, 70%, and 8.4 MPa, respectively. Following the incorporation of f-MWCNTs, these values changed to 3.37 MPa, 61%, and 15 MPa, respectively. These results indicate that the addition of f-MWCNTs significantly enhanced the tensile strength (σ) and Young’s modulus of the scaffold, while having no substantial effect on the strain at break.


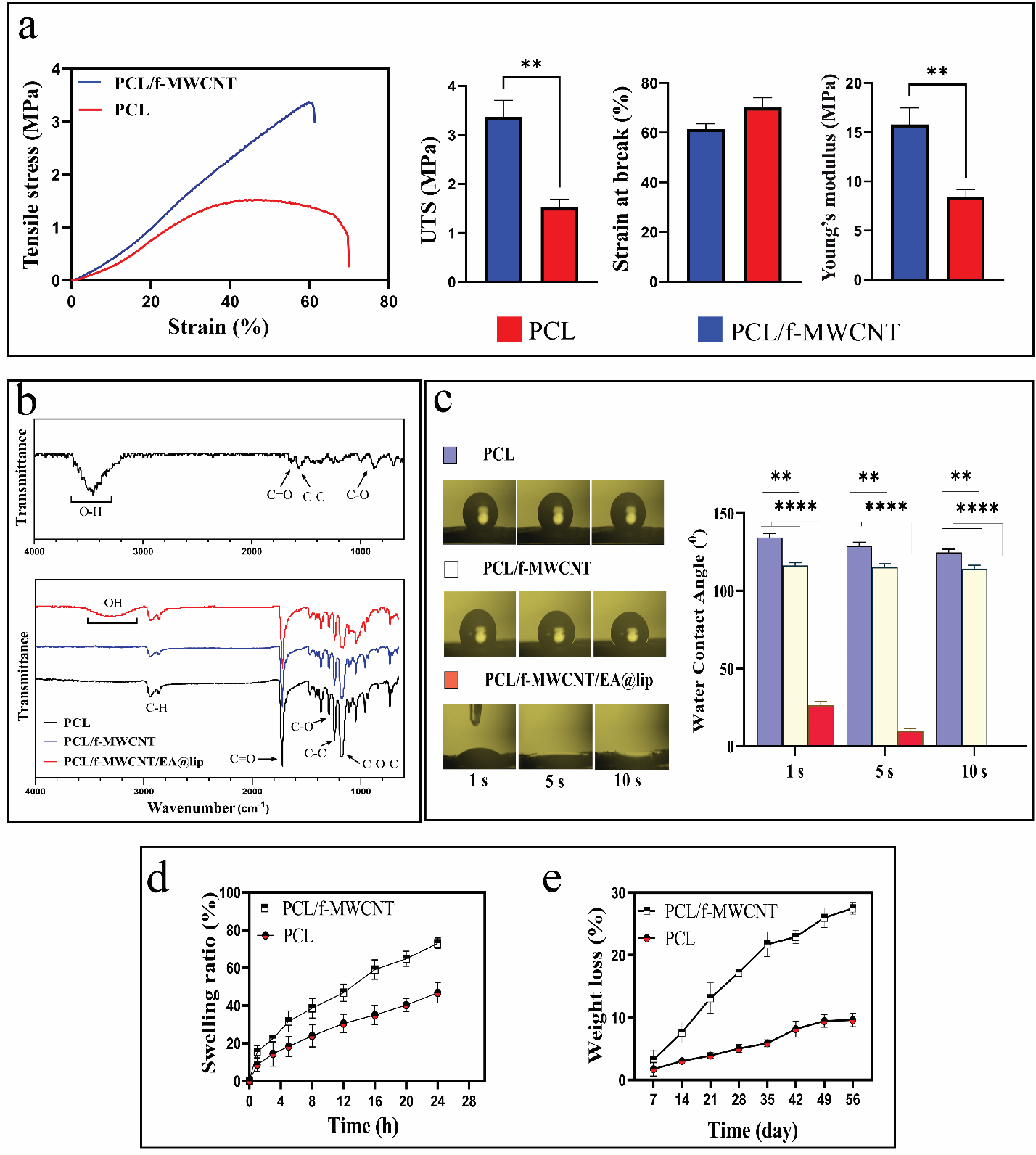


Figure S1. (a) Mean stress-strain curves obtained from five different samples of PCL and PCL/f-MWCNT electrospun scaffolds, ultimate tensile strength, strain at break, and Young's modulus obtained from five different samples of PCL and PCL/f-MWCNT. (b) FTIR spectra of f-MWCNT and ATR-FTIR spectra of PCL, PCL/f-MWCNT, and PCL/f-MWCNT/EA@lip scaffolds. (c) Water contact angle images and Comparison diagram of measured water contact angles at different times for the scaffolds. Data represented as Mean ± standard deviation (n = 3). Asterisks denote significant differences (*P < 0.05, **P < 0.01, and **** P < 0.0001). (d) Swelling ratio of PCL and PCL/f-MWCNT scaffolds at different times. (e) The addition of f-MWCNT to PCL has increased the weight loss of fibers by 2.8 times after 8 weeks.

Based on the FTIR spectrum of f-MWCNTs (Fig S1b), characteristic absorption peaks were observed for: C–C stretching at 1564 cm⁻¹, O–H stretching in the range of 3150–3650 cm⁻¹, C=O stretching at 1642 cm⁻¹, and C–O stretching at 874 cm⁻¹ (1,2). In the ATR-FTIR spectrum of PCL (Fig S1b), typical absorption bands included asymmetric –CH₂ stretching at 2940 cm⁻¹, symmetric –CH₂ stretching at 2864 cm⁻¹, carbonyl (C=O) stretching at 1723 cm⁻¹, C–O stretching at 1293 cm⁻¹, C–C stretching in the crystalline phase at 1240 cm⁻¹, asymmetric C–O–C stretching at 1174 cm⁻¹, and symmetric C–O–C stretching at 1159 cm⁻¹ (3). No new absorption peaks were detected after the incorporation of f-MWCNTs into the PCL matrix, indicating no chemical bond formation. However, the appearance of broad absorption bands in the 3100–3600 cm⁻¹ region in the ATR-FTIR spectrum of the PCL/f-MWCNT/EA@lip scaffold confirms the presence of EA-loaded liposomes on the scaffold surface, attributed to –OH stretching vibrations. Figure S1c presents the water contact angle (WCA) images at 1, 5, and 10 seconds, along with the corresponding angle measurements. The WCA for the PCL scaffold ranged from 133° to 123° across the time points. Upon adding f-MWCNTs, the angle decreased to approximately 116°–114°, indicating increased hydrophilicity. When EA@lip was incorporated, the WCA further dropped dramatically to 26°–0°, showing a significant enhancement in wettability. Compared to the PCL scaffold, the WCA of the PCL/f-MWCNT and PCL/f-MWCNT/EA@lip scaffolds decreased by approximately 12.8% and 80%, respectively, at t = 1 s. The swelling ratio (SR) of the scaffolds after 24 hours is shown in Fig S1d. The SR increased from 46.7 ± 5.4% for the PCL scaffold to 72.9 ± 2.8% for the PCL/f-MWCNT scaffold. The in vitro degradation of the scaffolds was assessed by measuring their weight loss over time, as shown in Fig S1e. After 8 weeks, the PCL/f-MWCNT scaffold exhibited 1.56-fold greater weight loss than the PCL scaffold (72% vs. 46%, respectively). Additionally, small fragmented pieces were observed in all scaffold samples after the 8-week degradation period.

**3.3. Improvement of biological and biochemical properties of scaffolds for ADMSCs through EA@lip covering**

We used drug dilution to determine the optimal concentration for viability and radical scavenging assays. The results showed that encapsulation of EA enhanced cell survival rates as the drug concentration increased. However, at higher concentrations, encapsulated EA exhibited greater cytotoxicity than free EA, as shown in Fig S2a. Moreover, encapsulated EA was more effective in eliminating free radicals at various time points (Fig S2a). Based on these findings, a concentration of 57.5 µg/ml was selected as the optimal dose for biological and biochemical analyses, based on its performance across multiple time points. The IC_50_ values of the tested compounds are summarized in Table S3. According to the results of both the DPPH and MTT assays, a concentration of 57.5 µg/ml of EA@lip demonstrated the highest free radical scavenging capacity on days 1, 3, and 7, without inducing cytotoxicity in ADMSCs. Therefore, this concentration was chosen for all subsequent experiments.

Table S3. DPPH radical scavenging and cytotoxicity activities of drugs for 1,3, and 7 d after treatment.

| Sample | \| DPPH radical scavenging  IC_50_ (µg/ml) \| \| --- \| \| \| D1 \| D3 \| D7 \| \| --- \| --- \| --- \| \| | \| Cytotoxicity activities IC_50_ (µg/ml) \| \| --- \| \| \| D1 \| D3 \| D7 \| \| --- \| --- \| --- \| \| |
| --- | --- | --- | --- | --- | --- | --- | --- | --- | --- | --- | --- | --- |
| EA | \| 112.1 ± 15.06 \| 58.2 ± 9.11 \| 48.3 ± 5.33 \| \| --- \| --- \| --- \| | \| 17.4 ± 0.1 \| 18.6 ± 0.93 \| 20.7 ± 1.03 \| \| --- \| --- \| --- \| |
| EA@lip | \| 163.6 ± 2.55 \| 109.3 ± 1.65 \| 136.2 ± 1.63 \| \| --- \| --- \| --- \| | \| 35.9 ± 3.1 \| 22.5 ± 1.05 \| 12.3 ± 0.97 \| \| --- \| --- \| --- \| |

Results presented as mean ± SD (n=3).

Fig S2b shows DAPI-stained cell nuclei to confirm the presence of viable cells attached to the scaffolds. Blue-stained nuclei represent live cells, and a higher density of blue signals indicates improved adhesion capacity. According to the DAPI results, blue-stained cells were observed across all scaffolds, suggesting that each scaffold supported initial cell attachment. However, the number of attached and viable ADMSCs was higher on the PCL/f-MWCNT scaffolds than on the pure PCL scaffold. The highest number of viable cells was detected on the PCL/f-MWCNT scaffold loaded with EA@liposomes. Cell proliferation was assessed by measuring the metabolic activity of ADMSCs at 1, 3, and 7 days (Fig S2c). On day 1, no significant differences in cell viability were observed among the groups. However, by days 3 and 7, both PCL/f-MWCNT and PCL/f-MWCNT/EA@lip scaffolds showed a significant increase in cell viability compared to the control and PCL-only groups (P < 0.05). Notably, the PCL scaffold alone exhibited the lowest viability at both time points. Overall, the MTT results demonstrated that PCL/f-MWCNT and PCL/f-MWCNT/EA@lip scaffolds supported significantly enhanced proliferation of ADMSCs compared to the control and PCL-only scaffolds. Fig S2d shows the morphology of cells on PCL, PCL/f-MWCNT, and PCL/f-MWCNT/EA@lip scaffolds. Cells exhibited elongated and well-spread morphologies across the nanofibrous substrates. In particular, extensive mesenchymal spreading and fibrillar ECM-like extensions were evident on the PCL/f-MWCNT and PCL/f-MWCNT/EA@lip scaffolds. Based on visual estimation, cell coverage area reached approximately 43%, 29%, and 4% on PCL/f-MWCNT/EA@lip, PCL/f-MWCNT, and PCL scaffolds, respectively. This corresponds to a 1.48-fold increase in surface coverage for PCL/f-MWCNT/EA@lip compared to PCL/f-MWCNT, and a 9.6-fold increase compared to PCL. The viability and DNA integrity of cells on the scaffolds were further validated using both MTT and DAPI staining assays. The hydrophilicity of the scaffolds played a critical role in enhancing cell attachment. As previously reported by Mansourpanah et al., the decrease in water contact angle (WCA) in PCL/carboxylated MWCNT composites is attributed to the presence of –COO⁻ functional groups on functionalized MWCNTs (4). In the current study, the incorporation of 3 wt% f-MWCNTs significantly improved both the hydrophilicity and swelling ratio (SR) of the nanofibers. FTIR analysis confirmed the presence of –COOH groups on the surface of the MWCNTs as a contributing factor. Furthermore, when ADMSCs were cultured on the PCL/f-MWCNT scaffold, the oxidative stress induced by H₂O₂ was markedly reduced. These cells exhibited suppressed ROS production and maintained TAC levels comparable to the control. However, complete suppression of LPO and full preservation of TTM was only achieved in the presence of EA@liposomes, highlighting the essential role of EA in reinforcing the scaffold's antioxidant capacity.

As demonstrated in Fig S2e, exposure of ADMSCs to H₂O₂ resulted in a significant increase in lipid peroxidation (LPO) levels compared with the control group (P < 0.0001). The PCL scaffold alone did not significantly reduce LPO levels compared to the H₂O₂-treated group. However, scaffolds containing carbon nanotubes (CNTs) significantly decreased LPO levels (P < 0.001 vs. H₂O₂ group), and the PCL/f-MWCNT/EA@lip scaffold further reduced LPO compared with the PCL/f-MWCNT group (P < 0.05). Regarding reactive oxygen species (ROS) levels, cells treated with H₂O₂ exhibited markedly elevated ROS compared with the control (P < 0.0001). The PCL/f-MWCNT scaffold significantly decreased ROS levels (P < 0.05 vs. H₂O₂ group), and a more pronounced reduction was observed in cells treated with the PCL/f-MWCNT/EA@lip scaffold compared to both the H₂O₂ (P < 0.0001) and PCL/f-MWCNT groups (P < 0.05). The effect of the scaffolds on total antioxidant capacity (TAC) is also shown in Fig S2e. H₂O₂ exposure significantly reduced TAC compared with the control (P < 0.0001), and the PCL-only scaffold did not restore antioxidant capacity. In contrast, scaffolds containing CNTs (PCL/f-MWCNT and PCL/f-MWCNT/EA@lip) significantly increased TAC, restoring it to levels comparable with the control group. Similarly, the level of total thiol molecules (TTM) was significantly decreased in H₂O₂-treated cells compared with the control group (P < 0.0001). Treatment with PCL/f-MWCNT and PCL/f-MWCNT/EA@lip scaffolds significantly increased TTM levels (P < 0.05 and P < 0.0001, respectively) compared with the H₂O₂ group. However, the PCL-only scaffold did not significantly improve TTM levels.


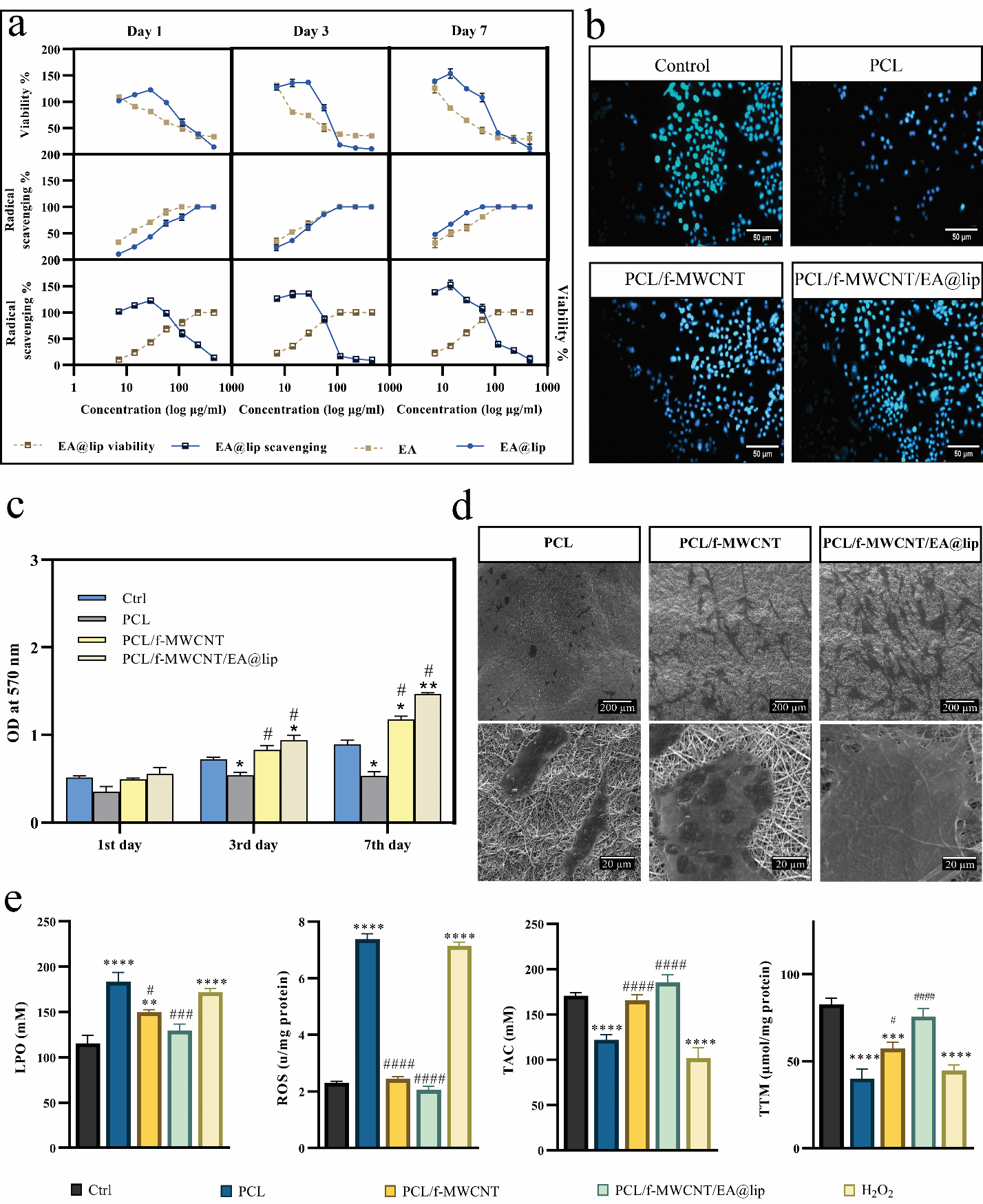


Figure S2. (a) Cell viability and antioxidant power changes versus the drug (EA) concentration after 1, 3, and 7 days of treatment. (b) Fluorescence microscope images of the DAPI staining after 3 days of ADMSCs cultured on samples. (d) FE-SEM images of ADMSCs cultured on PCL, PCL/f-MWCNT, and PCL/f-MWCNT/EA@lip scaffolds after 3 days. (c) Comparison of ADMSCs proliferation on the samples as determined by an MTT assay during 1, 3 and 7 days. Asterisk indicate significant differences from the control group and # indicates the difference from PCL scaffolds (* P < 0.05, ** P<0.01 and # P<0.05). (e) The effect of different scaffolds on lipid peroxidation (LPO), reactive oxygen species (ROS), total antioxidant capacity (TAC), and total thiol molecules (TTM). Asterisks (*) indicate significant differences from the control group and # indicates the difference from H_2_O_2_ (**P<0.001, ***P<0.001, ****P<0.0001, #P<0.05, ###P<0.001 and ####P<0.0001). Data represented as mean ± standard deviation (n = 3).

Reactive oxygen species (ROS), which arise from elevated OS, are highly reactive molecules capable of abstracting electrons from cellular macromolecules, thereby rendering them biologically inactive (5). Self-propagating free radical chain reactions mediate LPO and contribute to membrane damage (6). The accumulation of OS leads to the depletion of TTM and compromises the defensive capacity of TAC. Although findings remain somewhat inconsistent, there is evidence that CNT fibers can function as ROS scavengers (7). The results of our in vitro study demonstrated that the incorporation of functionalized MWCNTs (f-MWCNTs) into the scaffold under oxidative conditions significantly reduced MDA and ROS levels, while enhancing TTM and TAC levels in ADMSCs. The homogeneous distribution and uniform alignment of f-MWCNTs within the nanofibers are key factors ensuring the high antioxidant performance of the PCL/f-MWCNT composite scaffolds (8). Moreover, the physicochemical properties of CNTs can be tailored via covalent and non-covalent functionalization, allowing for the modification of surface charge, increased aqueous solubility, reduced agglomeration, and even the targeted delivery of anti-ROS agents (9). The electron affinity of CNTs and their capacity for radical addition to the sp²-hybridized carbon framework enable them to act as efficient free radical scavengers (10). Our findings indicate that the effective dispersion of f-MWCNTs within the PCL fibers—as confirmed by the uniform nanostructure of the electrospun scaffold and the non-agglomerated nanotubes observed in TEM images—imparts antioxidant capabilities to the scaffold by disrupting redox chain propagation, thus enhancing its therapeutic potential in oxidative environments.

**References**

1. Pavlidis I V, Tsoufis T, Enotiadis A, Gournis D, Stamatis H. Functionalized Multi-Wall Carbon Nanotubes for Lipase Immobilization. Adv Eng Mater [Internet]. 2010 May 1;12(5):B179–83. Available from: https://doi.org/10.1002/adem.200980021

2. Wu TM, Chen EC. Crystallization behavior of poly(ε-caprolactone)/multiwalled carbon nanotube composites. J Polym Sci Part B Polym Phys [Internet]. 2006 Feb 1;44(3):598–606. Available from: https://doi.org/10.1002/polb.20722

3. Zadehnajar P, Karbasi S, Akbari B, Ghasemi L. Incorporation of multi-walled carbon nanotubes into electrospun PCL/gelatin scaffold: the influence on the physical, chemical and thermal properties and cell response for tissue engineering. Mater Technol [Internet]. 2020 Jan 2;35(1):39–49. Available from: https://doi.org/10.1080/10667857.2019.1651539

4. Mansourpanah Y, Madaeni SS, Rahimpour A, Adeli M, Hashemi M, Moradian M. Fabrication new PES-based mixed matrix nanocomposite membranes using polycaprolactone modified carbon nanotubes as the additive: Property changes and morphological studies. Desalination. 2011;277:171–7.

5. Adibhatla RM, Hatcher JF. Lipid oxidation and peroxidation in CNS health and disease: from molecular mechanisms to therapeutic opportunities. Antioxid Redox Signal. 2010;12(1):125–69.

6. Lee YK, Kim SW, Park JY, Kang WC, Kang YJ, Khang D. Suppression of human arthritis synovial fibroblasts inflammation using dexamethasone-carbon nanotubes via increasing caveolin-dependent endocytosis and recovering mitochondrial membrane potential. Int J Nanomedicine. 2017;12:5761–79.

7. Tsuruoka S, Takeuchi K, Koyama K, Noguchi T, Endo M, Tristan F, et al. ROS evaluation for a series of CNTs and their derivatives using an ESR method with DMPO. In: Journal of Physics: Conference Series. IOP Publishing; 2013. p. 12029.

8. Liao GY, Zhou XP, Chen L, Zeng XY, Xie XL, Mai YW. Electrospun aligned PLLA/PCL/functionalised multiwalled carbon nanotube composite fibrous membranes and their bio/mechanical properties. Compos Sci Technol [Internet]. 2012;72(2):248–55. Available from: https://www.sciencedirect.com/science/article/pii/S0266353811004015

9. Dal Bosco L, Weber GE, Parfitt GM, Cordeiro AP, Sahoo SK, Fantini C, et al. Biopersistence of PEGylated carbon nanotubes promotes a delayed antioxidant response after infusion into the rat hippocampus. PLoS One. 2015;10(6):e0129156.

10. Eleftheriadou D, Kesidou D, Moura F, Felli E, Song W. Redox-Responsive Nanobiomaterials-Based Therapeutics for Neurodegenerative Diseases. Small [Internet]. 2020 Oct 1;16(43):1907308. Available from: https://doi.org/10.1002/smll.201907308
